# Metformin accelerates bone fracture healing by promoting type H vessel formation through inhibition of YAP1/TAZ expression

**DOI:** 10.1038/s41413-023-00279-4

**Published:** 2023-08-16

**Authors:** Zhe Ruan, Hao Yin, Teng-Fei Wan, Zhi-Rou Lin, Shu-Shan Zhao, Hai-Tao Long, Cheng Long, Zhao-Hui Li, Yu-Qi Liu, Hao Luo, Liang Cheng, Can Chen, Min Zeng, Zhang-Yuan Lin, Rui-Bo Zhao, Chun-Yuan Chen, Zhen-Xing Wang, Zheng-Zhao Liu, Jia Cao, Yi-Yi Wang, Ling Jin, Yi-Wei Liu, Guo-Qiang Zhu, Jing-Tao Zou, Jiang-Shan Gong, Yi Luo, Yin Hu, Yong Zhu, Hui Xie

**Affiliations:** 1 Department of Orthopedics, Xiangya Hospital, Central South University, Changsha, Hunan 410008 China; 2 Movement System Injury and Repair Research Center, Xiangya Hospital, Central South University, Changsha, Hunan 410008 China; 3 Hunan Key Laboratory of Angmedicine, Changsha, Hunan 410008 China; 4 Angmedicine Research Center of Central South University, Changsha, Hunan 410008 China; 5 The First Affiliated Hospital, Department of Metabolism and Endocrinology, Hengyang Medical School, University of South China, Hengyang, Hunan 421001 China; 6 National Clinical Research Center for Geriatric Disorders, Xiangya Hospital, Central South University, Changsha, Hunan 410008 China

**Keywords:** Bone quality and biomechanics, Bone

## Abstract

Due to increasing morbidity worldwide, fractures are becoming an emerging public health concern. This study aimed to investigate the effect of metformin on the healing of osteoporotic as well as normal fractures. Type H vessels have recently been identified as a bone-specific vascular subtype that supports osteogenesis. Here, we show that metformin accelerated fracture healing in both osteoporotic and normal mice. Moreover, metformin promoted angiogenesis in vitro under hypoxia as well as type H vessel formation throughout fracture healing. Mechanistically, metformin increased the expression of HIF-1α, an important positive regulator of type H vessel formation, by inhibiting the expression of YAP1/TAZ in calluses and hypoxia-cultured human microvascular endothelial cells (HMECs). The results of *HIF-1α* or *YAP1/TAZ* interference in hypoxia-cultured HMECs using siRNA further suggested that the enhancement of HIF-1α and its target genes by metformin is primarily through YAP1/TAZ inhibition. Finally, overexpression of YAP1/TAZ partially counteracted the effect of metformin in promoting type H vessel-induced angiogenesis-osteogenesis coupling during fracture repair. In summary, our findings suggest that metformin has the potential to be a therapeutic agent for fractures by promoting type H vessel formation through YAP1/TAZ inhibition.

## Introduction

Fractures are the most common traumatic injuries in humans and are mainly caused by traffic accidents, sports injuries, and medical conditions characterized by bone loss and bone destruction due to aging, menopause, or cancer.^1^ Fractures result in a heavy socioeconomic and medical burden. The prevalence of clinical fractures has reached 4% among people aged 40 years or older in China,^2^ while in the United States, the annual cost of osteoporotic fractures is projected to be approximately $25.3 billion by 2025.^3^

Fractures generally require surgical treatment in the clinical setting. Despite the remarkable regenerative capacity of bone, approximately 10%–15% of fracture patients suffer from delayed union or nonunion even with proper reduction and fixation.^4^ In particular, the presence of osteoporosis makes fracture surgery more challenging, and the additional immobility and prolonged healing time lead to higher rates of disability and mortality.^5^ Although the FDA has approved human bone morphogenetic protein (rhBMP-2) to accelerate fracture healing, the supraphysiologic dose required for osteoinduction makes this therapy expensive and may cause adverse effects, including ectopic ossification and immune reactions.^6^ In addition, some antiosteoporosis drugs, such as bisphosphonates, calcitonin, strontium ranelate, and parathyroid hormone, appear to facilitate fracture repair, but their long-term usage is also limited by the associated side effects.^7^ Hence, there remains an urgent need for pharmacological therapies to accelerate fracture healing.

Bone is a highly dynamic tissue in which angiogenesis and osteogenesis are closely linked during skeletal development, modeling, and repair.^8^ A specific vascular subtype in bone termed type H vessels, characterized by strong positivity for both CD31 and Endomucin (Emcn) in the endothelium, has recently been shown to couple angiogenesis with osteogenesis.^9,10^ Although the abundance of type H vessels is relatively limited, they preferentially associate spatially with perivascular osteoprogenitor cells and generate a specific niche that facilitates bone formation by providing angiocrine factors.^9^ Follow-up studies have identified HIF-1α (hypoxia-inducible factor 1-α),^9^ the Notch signaling pathway,^10^ platelet-derived growth factor-BB (PDGF-BB),^11^ and SLIT3 (slit guidance ligand 3)^12^ as important positive regulators of type H vessel-induced angiogenesis-osteogenesis coupling. The involvement of type H vessels throughout the regeneration of bone defects has also been identified.^13^ Therefore, induction of type H vessel angiogenesis in the local fracture region may be a new strategy to accelerate fracture healing.

The Hippo signaling pathway is evolutionarily conserved and plays an important role in the regulation of organ size, tissue regeneration, and cancer development.^14^ The transcriptional coactivator Yes-associated protein 1 (YAP1) and transcriptional coactivator with PDZ-binding motif (TAZ, also known as WWTR1), a key component of the Hippo signaling pathway, enter the nucleus in a nonphosphorylated state and interact with transcription factors (mostly the transcriptional enhancer associate domain family members) to induce downstream target gene expression and participate in cell proliferation, differentiation, and apoptosis.^15^ The proper regulatory effect of YAP1/TAZ signaling plays an essential role in sprouting angiogenesis, and endothelial-specific deletion of YAP1/TAZ leads to severely impaired retinal and cerebral vascularization.^16^ HIF-1α is a transcription factor that mediates the cellular response to altered oxygen levels and has been identified to be closely associated with physiological and pathological angiogenesis.^17^ However, a recent study found that YAP1/TAZ is a negative regulator of bone angiogenesis by suppressing HIF-1α target genes in bone endothelial cells,^18^ which suggested that drugs inhibiting endothelial YAP1/TAZ signaling may have a therapeutic effect by promoting bone angiogenesis.

Metformin is widely used in the treatment of insulin resistance-based diseases such as type II diabetes, metabolic syndrome, and nonalcoholic fatty liver disease.^19^ Recent studies have shown that metformin also has potential beneficial effects on bone tissue. Clinical trials have reported that metformin reduces the risk of fracture among diabetic patients compared to those taking other antidiabetic drugs.^20^ In vitro studies also found that metformin had direct osteogenic effects on osteoblasts and increased osteogenic induction of bone marrow progenitor cells.^21,22^ However, in vivo studies investigating the effects of metformin on nondiabetic bone repair are extremely limited, and the results are controversial.^21,23,24^ Furthermore, to the best of our knowledge, it is not yet clear whether metformin can regulate angiogenesis, particularly type H vessel formation, and endothelial YAP1/TAZ signaling during fracture healing.

In the current study, we found that metformin improved the function of endothelial cells under hypoxic conditions. We used mouse models to determine the bone regenerative potential of metformin in osteoporotic as well as normal fracture healing. Metformin accelerated the fracture healing process, and this promotion of osteogenic repair was associated with increased type H vessel angiogenesis, in part by inhibiting YAP1/TAZ and thus activating HIF-1α signaling in bone endothelial cells.

## Results

### Metformin promotes angiogenesis in vitro under hypoxic conditions

Fracture healing is a complicated process, and numerous studies have demonstrated that vascular function has an essential role in the repair process after fracture. To investigate the effect of metformin on angiogenesis during fracture healing, we first conducted in vitro experiments using human microvascular endothelial cells (HMECs). The local microenvironment at the fracture site is relatively hypoxic, so we cultivated HMECs under normoxic (21% O_2_) or hypoxic (1% O_2_) conditions and treated them with different concentrations of metformin (50, 250, and 500 μmol·L^−1^).

**Fig. 1:**
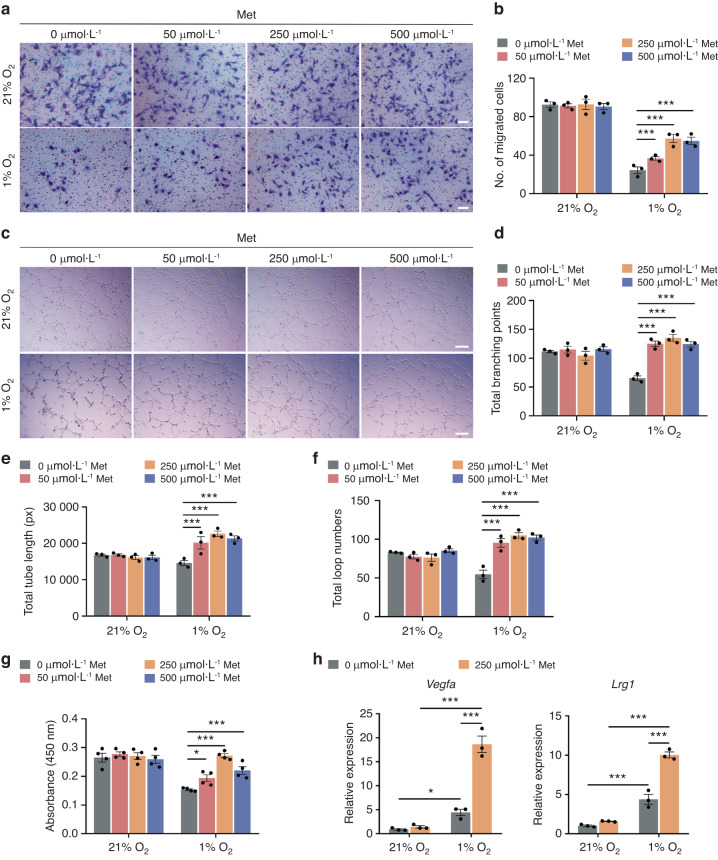
Metformin promotes angiogenesis under hypoxic conditions in vitro. Representative images (**a**) of the transwell migration assay with quantification of crystal violet-stained migrated HMECs (**b**) treated with metformin under different oxygen concentrations (21% and 1% O_2_). Met: metformin. Scale bar: 100 μm. *n* = 3 per group. Representative images (**c**) of tube formation of HMECs on Matrigel with quantification of the total branching points (**d**), total tube length (**e**), and total loop numbers (**f**). Scale bar: 200 μm. *n* = 3 per group. **g** CCK-8 analysis of the proliferation of HMECs. *n* = 4 per group. **h** qRT‒PCR analysis of the mRNA levels of the HIF-1α target genes *Vegfa* and *Lrg1* in HMECs with or without metformin treatment under different oxygen concentrations. *n* = 3 per group. Data are presented as the mean ± SEM. ^*^P < 0.05, ^**^P < 0.01, and ^***^P < 0.001

Relative to normoxia, hypoxia resulted in impaired migration (Fig. 1a, b), tube formation (Fig. 1c–f), and proliferation (Fig. 1g) of HMECs. However, metformin administration substantially augmented the reproduction and functions of HMECs under hypoxic but not normoxic conditions (Fig. 1a–g). In addition, HMECs cultured under hypoxic conditions dramatically upregulated the expression of the HIF-1α target genes *Vegfa* and *Lrg1* compared to their expression under normoxic conditions. This expression of *Vegfa* and *Lrg1* was further amplified after treatment with metformin under hypoxic conditions (Fig. 1h). These results indicated that metformin improved HMEC function in vitro under hypoxia.

### Metformin accelerates bone fracture healing in osteoporotic and normal mice

**Fig. 2:**
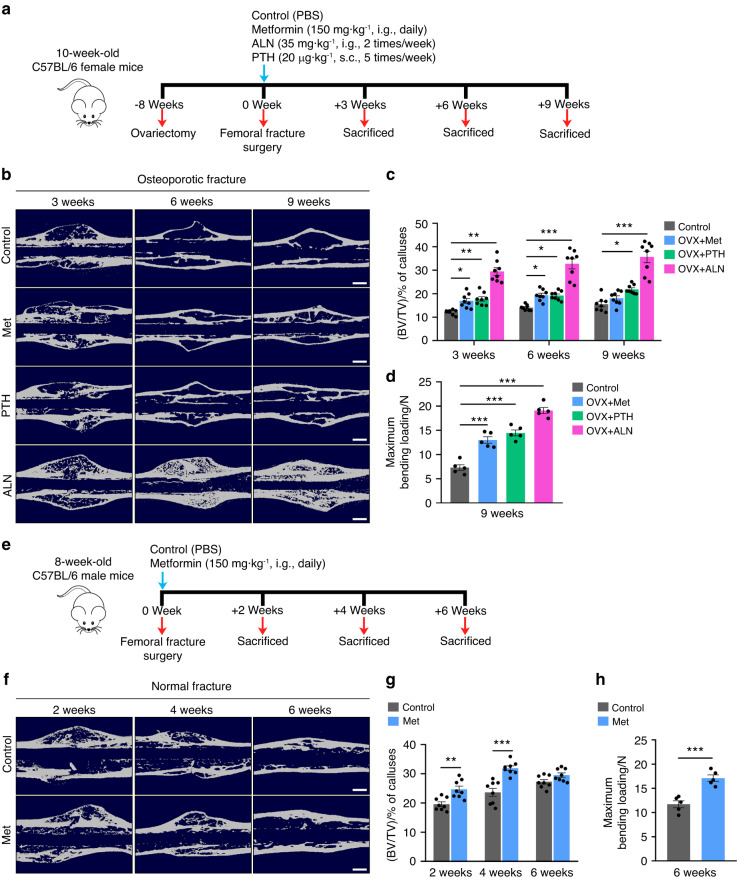
Metformin accelerates bone fracture healing in osteoporotic and normal mice. **a** Schematic diagram of the experimental design for assessing the effects of metformin on fracture healing in osteoporotic mice. **b** Representative μCT reconstruction images of fractured femora from the osteoporotic mice treated with PBS, Met, PTH, and ALN at 3, 6, and 9 weeks post-fracture. Met metformin, ALN alendronate, PTH parathyroid hormone. Scale bar: 60 μm. **c** Quantitative analysis of the bone volume fraction (BV/TV) of calluses during osteoporotic fracture healing. *n* = 8 per group. **d** Three-point bending test of femoral ultimate load at 9 weeks post-osteoporotic fracture. *n* = 5 per group. **e** Schematic diagram of the experimental design for assessing the effects of metformin on fracture healing in normal mice. **f** Representative μCT reconstruction images of fractured femora from the normal mice treated with PBS and Met at 2, 4, and 6 weeks post-fracture. Scale bar: 60 μm. **g** Quantitative analysis of the bone volume fraction (BV/TV) of calluses during normal fracture healing. *n* = 8 per group. **h** Three-point bending test of femoral ultimate load at 6 weeks post-normal fracture. *n* = 5 per group. Data are presented as the mean ± SEM. ^*^P < 0.05, ^**^P < 0.01, and ^***^P < 0.001

Next, we constructed osteoporotic and normal fracture models to explore the effect of metformin on the fracture healing process. Closed fracture surgery and intramedullary fixation were performed on 18-week-old female mice that had undergone OVX surgery for 2 months (Fig. 2a), as well as on normal 8-week-old male mice (Fig. 2e). Given that impaired bone formation and remodeling delayed the healing process of osteoporotic mice, the animals were sacrificed at 3, 6, and 9 weeks post-operation for examination compared with normal mice (sacrificed at 2, 4, and 6 weeks post-operation).

The effects of metformin on bone fracture healing were indicated by µCT analysis and mechanometry of fractured bones. For the microarchitecture of the callus, the image of µCT reconstruction showed that more mineralized calluses were formed in the metformin-treated groups than their respective control groups at the early stage after fracture, regardless of whether osteoporotic (Fig. 2b) or normal mice were used (Fig. 2f). Consistently, from μCT data analysis, a significantly higher BV/TV of calluses was found in the mice receiving metformin in the early to middle stages of fracture healing (at weeks 3 and 6 for osteoporotic fractures and at weeks 2 and 4 for normal fractures, respectively) compared to their corresponding control mice (Fig. 2c, g). PTH and ALN are the most commonly used drugs for the prevention of osteoporosis. In the present study, we also compared their effects on the healing process of osteoporotic fractures. The image of µCT reconstruction showed that both PTH and ALN treatment accelerated osteoporotic fracture repair (Fig. 2). Consistently, the BV/TV of the callus was significantly increased throughout the healing process of osteoporotic fractures (Fig. 2c). These differences observed by µCT analysis were further verified by three-point bending tests, and flexural rigidity of the fractured femora at the endpoint of fracture healing (at week 9 for osteoporotic fractures and at week 6 for normal fractures) was significantly enhanced in the mice treated with metformin, PTH, and ALN (Fig. 2d, h).

Following the μCT data, H&E staining showed that both osteoporotic and normal mice with fracture exhibited more mineralized bone in calluses after metformin treatment compared to that of the control group (Fig. S1a, b). In addition, we used Alcian blue staining to assess the cartilage level in each group. As illustrated in Fig. S1c–f, metformin significantly increased the cartilage area of the callus in the early stage of fracture (3 weeks after osteoporotic fracture and 2 weeks after normal fracture) compared with that of the mice treated with vehicle. Concurrently, ALN substantially enhanced the proportion of cartilage in the callus at 3 and 6 weeks after osteoporotic fracture (Fig. S1c, e).

Furthermore, at the endpoint of fracture healing, ELISAs revealed augmented bone marrow OCN levels in the osteoporotic mice receiving metformin or PTH and elevated serum/bone marrow OCN levels in the normal mice receiving metformin compared to their PBS-treated controls (Fig. S2a, b). Interestingly, the number of adipocytes in the calluses was markedly reduced in both osteoporotic and normal mice in the metformin-treated group (Figure S2c, d). Moreover, to verify whether metformin directly promotes osteogenesis, we extracted mouse primary BMSCs and determined the effects of metformin on their osteogenic and adipogenic differentiation. Alizarin Red S and Oil Red O staining indicated that metformin at all concentrations (50, 250, and 500 μmol·L^−1^) induced significantly more calcium nodules (Fig. S2e, f) and less lipid droplet formation (Fig. S2g, h) than those of their respective controls. These results indicated that metformin exerted positive effects on osteogenesis both in vivo and in vitro.

### Metformin accelerates bone fracture healing by increasing type H vessel formation

**Fig. 3:**
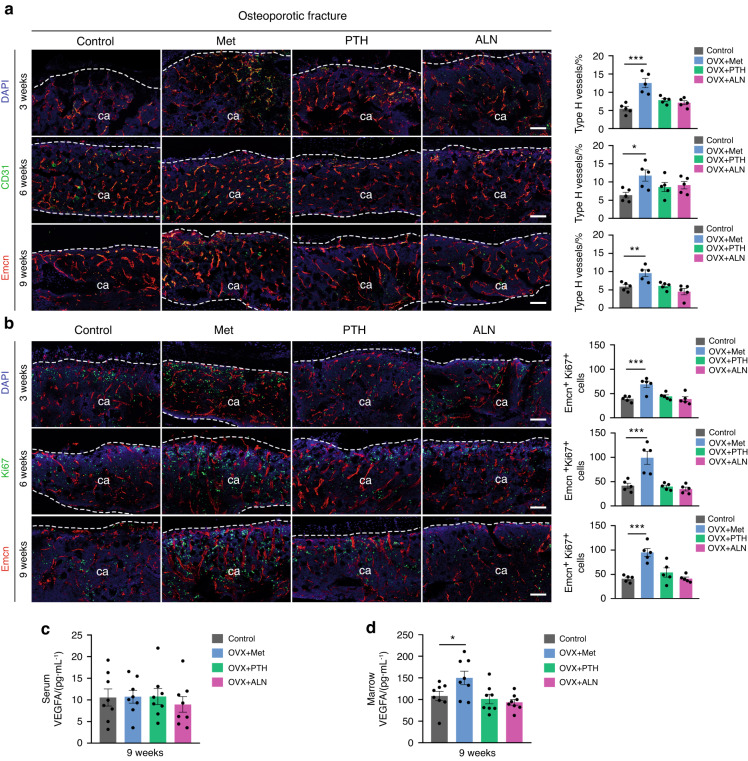
Metformin promotes type H vessel formation in osteoporotic fracture mice. **a** Representative CD31 and Emcn coimmunostaining images (left) with quantification of the type H vessel ratio in calluses (right) from the osteoporotic mice treated with PBS, Met, PTH, and ALN at 3, 6, and 9 weeks post-fracture. ca: callus. The dotted line represents the boundary of the callus. Met metformin, ALN alendronate, PTH parathyroid hormone. Scale bar: 100 μm. *n* = 5 per group. **b** Representative Ki67 and Emcn coimmunostaining images (left) with quantification of the number of Ki67-positive endothelial cells in calluses (right) from the osteoporotic mice treated with PBS, Met, PTH, and ALN at 3, 6, and 9 weeks post-fracture. Scale bar: 100 μm. *n* = 5 per group. ELISAs for the serum (**c**) and bone marrow (**d**) concentrations of VEGFA at 9 weeks post-osteoporotic fracture. *n* = 8 per group; Data are presented as the mean ± SEM. ^*^P < 0.05, ^**^P < 0.01, and ^***^P < 0.001

To investigate whether the metformin-promoted fracture repair process was accompanied by an increased expansion of type H vessels, we performed immunofluorescence staining. Notably, CD31 and Emcn coimmunostaining revealed that the formation of type H vessels was effectively enhanced in calluses throughout the repair process of both osteoporotic (Fig. 3a) and normal (Fig. S3a, b) fractures in the metformin-treated mice relative to those in their controls. Additionally, the numbers of proliferating endothelial cells in the fracture area, as reflected by Emcn^+^ Ki67^+^ immunostaining, were significantly increased at all time points after fracture in the metformin-treated group (Fig. 3b and Fig. S3c, d). In addition, ELISAs revealed that administration of metformin significantly increased VEGFA levels in bone marrow without detectable changes in serum (Fig. 3c, d and Fig. S3e, f). In contrast, the expression level of type H vessels, the number of proliferating endothelial cells, and the serum/bone marrow VEGFA concentration did not change in the PTH and ALN groups compared to the control group (Fig. 3a, b). These results suggested that enhanced type H vessel formation may be an important mechanism by which metformin facilitates fracture repair.

### Metformin inhibits the expression of YAP1/TAZ and promotes the expression of HIF-1α in HMECs under hypoxic conditions

**Fig. 4:**
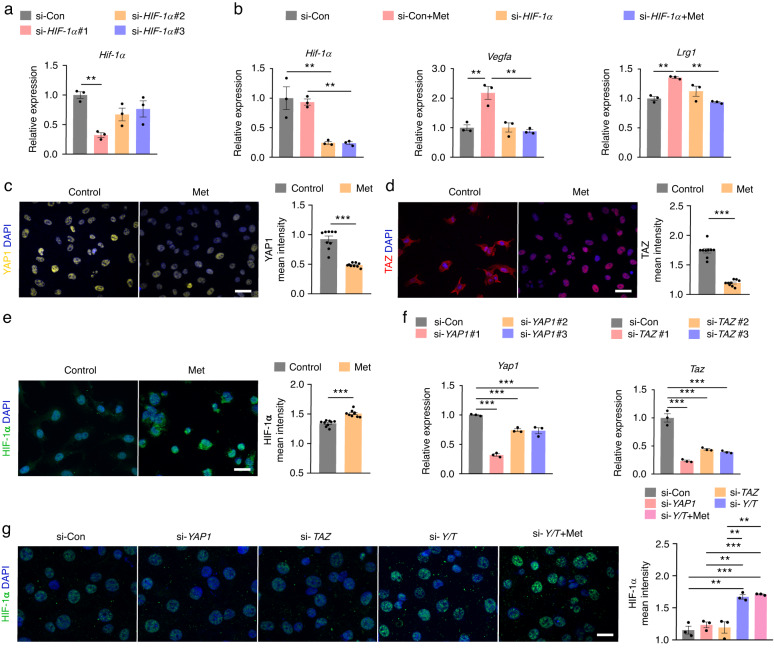
Metformin promotes the expression of HIF-1α by inhibiting the expression of YAP1/TAZ in HMECs under hypoxic conditions. **a** qRT‒PCR analysis of the inhibitory efficiency of siRNAs targeting *HIF-1α*. *n* = 3 per group. **b** qRT‒PCR analysis of the expression of *HIF-1α* and its target genes *Vegfa* and *Lrg1* in the si-*HIF-1α*-transfected HMECs with or without metformin treatment under hypoxic conditions (1% O_2_). Met: metformin. *n* = 3 per group. Immunofluorescence staining images and quantification showing the protein levels of HIF-1α (**c**), YAP1 (**d**), and TAZ (**e**) in the HMECs treated with PBS (control) or metformin under hypoxic conditions. Scale bar: 20 μm. *n* = 9 per group. **f** qRT‒PCR analysis of the inhibitory efficiency of siRNAs targeting *YAP1* or *TAZ*. *n* = 3 per group. **g** Immunofluorescence staining images and quantification showing the protein level of HIF-1α in the hypoxia-cultured HMECs from the si-Con, si-*YAP1*, si-*TAZ*, si-*Y/T*, and si-*Y/T* + Met groups. Y/T: YAP1 and TAZ. Scale bar: 20 μm. *n* = 3 per group. Data are presented as the mean ± SEM. ^*^P < 0.05, ^**^P < 0.01, and ^***^P < 0.001

Previous studies have shown that endothelial HIF-1α is a key regulatory signal for coupling angiogenesis and osteogenesis. We then examined whether the promotion of endothelial cell function by metformin under hypoxia was HIF-1α signaling dependent. We used siRNA to interfere with *HIF-1α* expression in hypoxia-cultured HMECs. si-*HIF-1α* #1 had the highest silencing efficiency and was selected for the following experiment (Fig. 4a). HIF-1α expression and that of the downstream genes *Vegfa* and *Lrg1* were examined after exposure to metformin. RT‒qPCR results showed that transcription of *HIF-1α* and these two proangiogenic genes could no longer be increased by metformin after *HIF-1α* knockdown (Fig. 4b).

Recently, Sivaraj et al. reported that YAP1/TAZ, a key component of the Hippo pathway, is a negative regulator of bone angiogenesis, which could coimmunoprecipitate with HIF-1α under hypoxic conditions and repress the transcription of its downstream genes.^18^ The role of metformin in regulating the Hippo pathway in the field of cancer has been extensively studied; however, whether it can modulate YAP1/TAZ signaling in endothelial cells has not been reported. To investigate this, we performed immunofluorescence analysis in vitro. Immunofluorescence staining of HMECs showed strong expression of YAP1 and TAZ in the nucleus under hypoxia; however, metformin treatment induced significantly less intranuclear expression of YAP1 and TAZ relative to the control (Fig. 4c, d). Importantly, HIF-1α expression was significantly increased in these endothelial cells (Fig. 4e), which was consistent with the increased expression of *Vegfa* and *Lrg1* downstream of *HIF-1α* (Fig. 1h).

We further used siRNA to interfere with *YAP1* and *TAZ* expression in hypoxia-cultured HMECs. As shown in Fig. 4f, si-*YAP1* #1 and si-*TAZ* #1 are the sequences with the highest silencing efficiency, and they were chosen for the following experiment. Immunofluorescence results showed that individual knockdown of *YAP1* or *TAZ* failed to alter HIF-1α expression; however, it was significantly augmented in the case of *YAP1* and *TAZ* double knockdown, and metformin did not further increase this effect (Fig. 4g). These results suggested that metformin upregulates HIF-1α and its target gene expression mainly by inhibiting YAP1/TAZ in HMECs under hypoxic conditions.

### Metformin promotes the expression of HIF-1α by inhibiting the expression of YAP1/TAZ during fracture healing

**Fig. 5:**
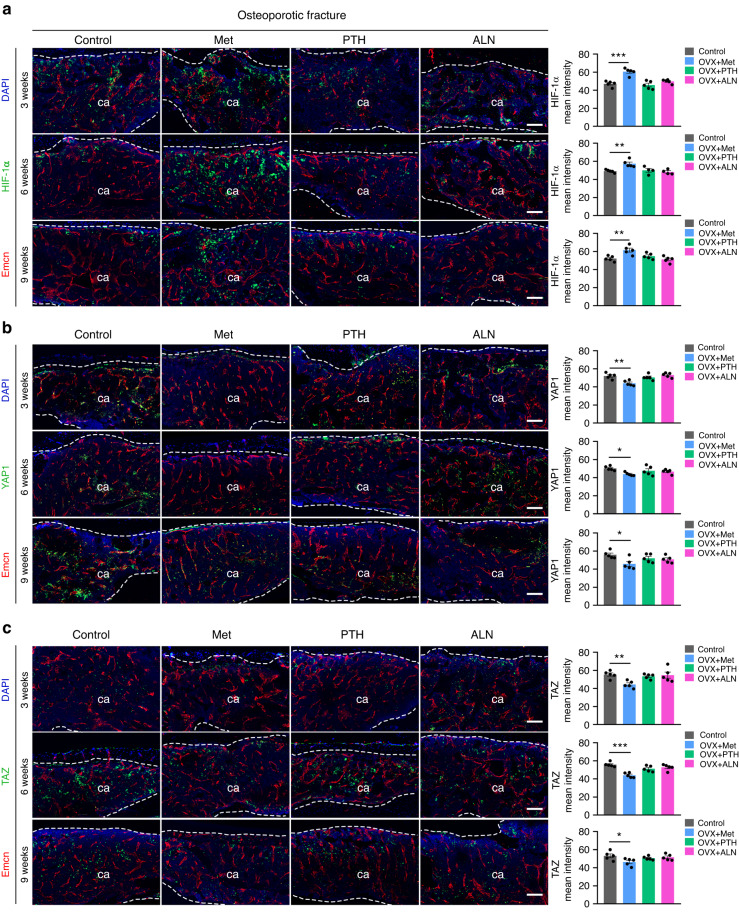
Metformin promotes HIF-1α expression by inhibiting YAP1/TAZ expression during osteoporotic fracture healing. **a** Representative HIF-1α and Emcn coimmunostaining images (left) with quantification (right) of the HIF-1α mean intensity in calluses from the osteoporotic mice treated with PBS, Met, PTH, and ALN at 3, 6, and 9 weeks post-fracture. ca: callus. The dotted line represents the boundary of the callus. Met metformin, ALN alendronate, PTH parathyroid hormone. Scale bar: 100 μm. *n* = 5 per group. **b** Representative YAP1 and Emcn coimmunostaining images (left) with quantification (right) of the YAP1 mean intensity in calluses from the osteoporotic mice at 3, 6, and 9 weeks post-fracture. Scale bar: 100 μm. *n* = 5 per group. **c** Representative TAZ and Emcn coimmunostaining images (left) with quantification (right) of the TAZ mean intensity in calluses from the osteoporotic mice at 3, 6, and 9 weeks post-fracture. Scale bar: 100 μm. *n* = 5 per group. Data are presented as the mean ± SEM. ^*^P < 0.05, ^**^P < 0.01, and ^***^P < 0.001

We further investigated whether metformin could also modulate YAP1/TAZ and thus HIF-1α signaling during fracture healing. Consistent with the in vitro experiments, immunostaining confirmed that the administration of metformin inhibited YAP1/TAZ expression in calluses and, conversely, significantly increased the expression of HIF-1α throughout the osteoporotic (Fig. 5a) and normal (Fig. S4) fracture repair. In contrast, there was no obvious alteration of YAP1/TAZ or HIF-1α expression in the healing regions of fractured bones in the osteoporotic mice treated with PTH and ALN (Fig. 5). In addition, immunostaining showed that metformin treatment significantly increased the expression of VEGFA and LRG1 in calli relative to that of the PBS-treated osteoporotic (Fig. S5) and normal mice (Fig. S6).

We also explored whether metformin could induce changes in the expression of the HIF-1α target gene *Vegfa* in osteoblast progenitors or bone marrow-derived macrophages (BMMs), thereby indirectly influencing angiogenesis. As revealed in Fig. S7a, c, *Vegfa* expression was significantly increased in MC3T3-E1 cells and BMMs under hypoxic conditions compared to normoxic conditions, while it was significantly decreased in BMSCs (Fig. S7b). Notably, metformin caused a marked reduction in *Vegfa* expression in these cells under hypoxic conditions but not under normoxic conditions (Fig. S7a, c). We then cocultured HMECs with the previously mentioned cells separately to determine whether metformin could indirectly affect the function of ECs. As shown in Fig. S7d–f, the migration of HMECs was not affected by either MC3T3-E1 cells or BMSCs themselves or by these cells treated with metformin. However, the number of migrated HMECs was significantly reduced in the BMM and metformin-treated BMM groups compared to the control group. These findings suggest that metformin enhances angiogenesis under hypoxic conditions primarily by directly affecting EC function.

### YAP1/TAZ overexpression impairs the promoting effect of metformin on osteoporotic fracture repair

**Fig. 6:**
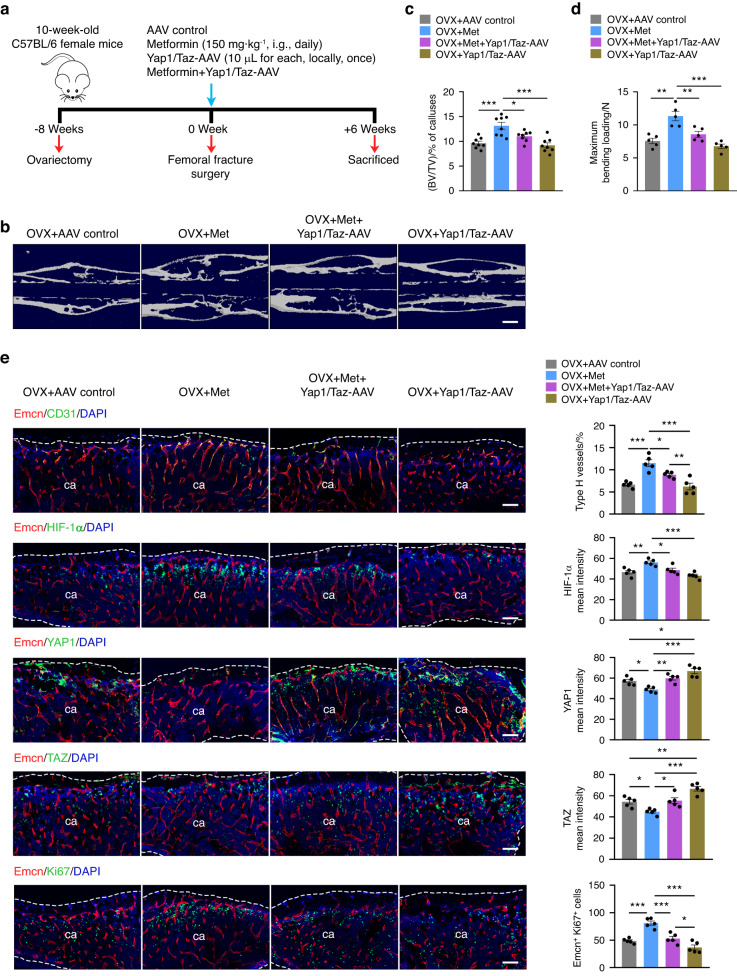
YAP1/TAZ overexpression impairs the promoting effect of metformin on osteoporotic fracture repair. **a** Schematic diagram of the experimental design for assessing the effects of metformin on fracture healing in the osteoporotic mice with YAP1/TAZ overexpression. **b** Representative μCT reconstruction images of fractured femora from the osteoporotic mice treated with the AAV control, Met, Met + Yap1/Taz-AAV, and Yap1/Taz-AAV at 6 weeks post-fracture. Met: metformin. Bar: 60 μm. **c** Quantitative analysis of the bone volume fraction (BV/TV) of calluses at 6 weeks post-osteoporotic fracture. *n* = 9 per group. **d** Three-point bending test of the femoral ultimate load at 6 weeks post-osteoporotic fracture. *n* = 5 per group. **e** Representative images of CD31, HIF-1α, YAP1, TAZ, and Ki67 coimmunostaining with Emcn in calluses from the osteoporotic mice at 6 weeks post-fracture (left) and their quantification analysis (right). ca: callus. The dotted line represents the boundary of the callus. Scale bar: 100 μm. *n* = 5 per group. Data are presented as the mean ± SEM. ^*^P < 0.05, ^**^P < 0.01, and ^***^P < 0.001

Given these results, we next investigated whether the overexpression of YAP1/TAZ would impair the effect of metformin in promoting osteoporotic fracture healing (Fig. 6a). We injected Yap1/Taz-overexpressing AAV and control AAV locally into the fracture area and re-evaluated the effect of metformin on osteoporotic fracture repair. Osteoporotic mice that underwent fracture surgery were divided into four groups: the AAV control group, metformin group, metformin plus Yap1/Taz-AAV group, and Yap1/Taz-AAV group.

Expression of the control AAV in the callus was detectable 6 weeks after injection, indicating that local injection of AAV2 has a reliable effect on gene expression in the fracture healing area (Fig. S8). The subsequent μCT examination showed that Yap1/Taz-AAV administration substantially countered the metformin-induced increase in mineralized bone of calluses, as indicated by a decreased BV/TV of calluses (Fig. 6b, c), which was accompanied by reduced biomechanical parameters of fractured bones (Fig. 6d). More importantly, immunofluorescence analysis revealed that markedly increased expression of YAP1/TAZ significantly reduced HIF-1α expression in the region of fracture healing and hampered type H vessel formation in the metformin group (Fig. 6e). The results suggested that the overexpression of YAP1/TAZ partially counteracted the facilitative effects of metformin on osteoporotic fracture recovery.

## Discussion

With increasing morbidity worldwide, fractures are becoming an emerging public health concern. Although extensive efforts have been made to explore effective medications for the prevention and treatment of fractures, new strategies are urgently needed for the development of anabolic drugs. Here, we found that in addition to direct osteogenic effects, metformin accelerated fracture healing by promoting type H vessel formation in both osteoporotic and normal mice by enhancing HIF-1α expression through inhibition of YAP1/TAZ signaling in endothelial cells. This finding may broaden the potential clinical applications of metformin and provide a new therapeutic strategy for the treatment of bone fractures.

Metformin has a variety of biological activities as a first-line treatment for type 2 diabetes mellitus, and its new antiaging and antitumor functions have been gradually recognized in recent years.^25^ Interestingly, a growing number of studies have suggested that metformin also has a role in regulating bone metabolism.^26^ Several recent meta-analyses of observational studies have indicated that metformin is associated with a reduced risk of fracture among patients with diabetes.^27,28^ Although the exact mechanism is still not fully understood, accumulating evidence suggests that AMPK is one of the main signaling pathways stimulated by metformin in bone, thereby tilting the differentiation balance of bone marrow mesenchymal stem cells toward osteoblastogenesis and promoting the proliferation and differentiation of osteogenic lineage cells.^22,29^ Studies using animal models to explore the effects of metformin on bone lesion repair are limited. Molinuevo et al. demonstrated that metformin treatment stimulated bone healing in a cranial defect model in both diabetic and nondiabetic rats.^21^ Similarly, Sedlinsky et al. found that metformin treatment increased the ossification of minimal parietal lesion repair in rats and prevented the inhibitory effects of rosiglitazone monotherapy.^23^ Nevertheless, another study by Jeyabalan et al. reported no significant effect of metformin on transverse osteotomy healing in Wistar rats.^24^ These controversial results might be derived from differences in the methodology, therapeutic concentration, and duration of metformin. In this study, we systematically investigated the role of metformin in closed femoral fracture healing and found that metformin accelerated repair in both osteoporotic and normal mice, as evidenced by the higher BV/TV values of calluses in the early and middle stages and better mechanical strength of the fractured bone at the endpoint. Numerous preclinical and clinical studies have explored the effect of bisphosphonates and PTH, classic antiosteoporotic drugs, on osteoporotic fracture healing. Bisphosphonate treatment was associated with larger callus development as well as improved final mechanical strength in osteoporotic rats.^30^ In addition, two randomized controlled trials have shown that intermittent PTH treatment accelerates the healing of the distal radius and pelvis in postmenopausal women.^31,32^ Our results are consistent with those of previous studies confirming that intermittent administration of ALN and PTH accelerated osteoporotic fracture healing.

The skeletal system has a highly vascularized network that not only delivers necessary nutrients and oxygen but also secretes specific cytokines that regulate a range of biological activities. The lack or inhibition of angiogenesis in the callus has been reported to be one of the main causes of delayed union or nonunion of fractures.^33^ The effect of metformin on angiogenesis varies in different organs or physiopathological conditions. On the one hand, studies have shown that metformin could enhance angiogenesis to promote poststroke recovery, postischemic limb perfusion, and wound healing in diabetic mice.^34–36^ On the other hand, metformin has been reported to suppress excessive angiogenesis to improve polycystic ovary syndrome and prevent multiple tumor growth and metastasis.^37,38^ However, the influence of metformin on angiogenesis during fracture healing has not been investigated. To achieve this, we first conducted in vitro experiments to examine the effect of metformin on HMECs in a hypoxic state, as this condition is more representative of the environment surrounding the fracture site after the disruption of the vascular supply.^39^ We found that metformin improved various steps of angiogenesis under hypoxia, including proliferation, migration, and tube formation of HMECs. Consistently, the number of proliferating endothelial cells during fracture healing was increased in the mice treated with metformin.

Type H vessels have recently been identified as tissue-specific vessels in bone and are characterized by high expression of endothelial CD31 and Emcn, which are mainly located adjacent to the growth plate in the metaphysis and along the endosteum of the diaphysis.^9^ This capillary subtype maintains bone homeostasis by mediating local angiogenesis and providing niche signals for perivascular osteoprogenitors, a process known as “angiogenesis-osteogenesis coupling”.^10^ Accumulating evidence underscores the important role of type H vessels required for bone modeling and remodeling.^40^ Indeed, pharmacological treatments such as recombinant SLIT3 or ophiopogonin D are effective in accelerating fracture healing by increasing the abundance of type H vessels in mouse fracture models.^12,41^ Our previous study also found that gastrointestinal administration of *A. muciniphila* stimulated this vascular subtype and improved bone repair.^13^ In this study, we observed that metformin increased the expansion of type H vessels throughout osteoporotic as well as normal fracture repair, which is a new mechanism by which metformin regulates bone metabolism independently of its direct osteogenic stimulating effect.

When injured, bone is locally encapsulated by hematoma, and a hypoxic environment is created, leading to a significant upregulation of HIF-1α and its downstream targets, such as VEGF, to stimulate angiogenesis and osteogenesis.^42^ Our in vitro results revealed that HIF-1α is critical for mediating metformin-promoted vascular function, as metformin could no longer increase the transcript levels of the proangiogenic genes *Vegfa* and *Lrg1* after *Hif-1α* knockdown in HMECs. Notably, Kusumbe et al. demonstrated that endothelial-specific HIF-1α plays a crucial role in the regulation of type H vessel formation.^9^ Inducible endothelial deletion of *Hif-1α* was associated with impaired type H vessel angiogenesis and bone formation, whereas stabilization of HIF-1α in the endothelium by targeting von Hippel‒Lindau tumor suppressor (*Vhl*) enhanced their expansion in metaphysis.^9^ Here, by immunofluorescence staining, we found that metformin significantly increased the expression of HIF-1α in HMECs under hypoxic conditions and consistently in calluses throughout fracture healing, which explains the augmented expansion of type H vessels by metformin in the fracture repair process.

As a major effector in the Hippo signaling pathway, the transcriptional coactivator YAP1/TAZ plays a critical role in development and tumor angiogenesis.^43^ Although the exact mechanism remains unclear, a recent study reported that YAP1/TAZ affects angiogenesis in the skeleton in a manner opposite to that of other organs.^18^ Postnatal endothelial cell-specific double knockout of *Yap1/Taz* reduced retinal angiogenesis but increased vascular growth, HIF-1α pathway target gene expression, and bone formation in long bones.^18^ Previous studies have reported that metformin, a classic AMPK agonist, reduces tumorigenicity in mouse embryonic fibroblasts by increasing YAP1/TAZ phosphorylation and decreasing the expression of YAP1 target genes.^44^ In addition, metformin can reduce hepatocellular carcinoma cell proliferation by directly phosphorylating YAP1 and reducing its expression through an AMPK-dependent mechanism.^45^ Consistent with these studies, we found that metformin inhibited YAP1/TAZ expression in HMECs under hypoxia as well as in calluses during fracture healing. However, local injection of Yap1/Taz-AAV enhanced their expression in calluses and counteracted metformin-accelerated osteoporotic fracture healing, as evidenced by the reduced bone volume fraction of calluses and biomechanical strength of fractured bones. Mechanistically, increased YAP1/TAZ accumulation reduced HIF-1α expression and subsequent type H vessel formation, as further evidenced by a significant increase in HIF-1α expression after the knockdown of YAP1/TAZ in HMECs under hypoxic conditions. In contrast, the pro-osteogenic effect of ALN and PTH appeared to be independent of their effects on YAP1/TAZ as well as HIF-1α expression. ALN was reported to enhance blood flow and type H capillaries in aged mice,^46^ while intermittent PTH administration in adult mice reduced the type H cell population.^47^ Here, we did not observe that the expansion of type H vessels was affected by ALN or PTH during osteoporotic fracture healing, which remains to be further investigated.

Although the current study found that metformin accelerated fracture healing, there are still some issues that need to be discussed. First, it is well known that the commitment of BMSCs to the osteoblast and adipocyte lineages is competitive.^48^ Our findings are consistent with previous reports verifying that metformin enhanced the osteogenic differentiation and inhibited the adipogenic differentiation of BMSCs,^21,49^ yet the current study did not explore the effect of metformin on osteoclast lineage cells that are also closely involved in bone regeneration. Second, YAP/TAZ are key effectors of the Hippo signaling pathway and are widely expressed across various cell types. In bone tissue, several studies have reported that YAP/TAZ can be expressed in hematopoietic stem cells (HSCs), BMSCs, ECs, osteoblasts and osteoclasts.^50–54^ Further research is needed to determine which specific cells within the callus express YAP/TAZ and whether these cells differ from those that can express YAP/TAZ in normal bone tissue. Similarly, the effect of metformin on ECs differs from that on MC3T3-E1 cells, BMSCs and BMMs, which may be due to a dose‒response issue; this discrepancy also requires further investigation. Finally, we only investigated the effect of metformin on postmenopausal estrogen-deficient osteoporotic fractures, and it would also be of interest to determine whether metformin, a classic antidiabetic and promising antiaging drug, has a role in diabetic or senile osteoporotic fracture healing.

In summary, our findings demonstrate that metformin increases type H vessels and bone formation during fracture healing. The underlying mechanism may be that metformin promotes angiogenesis in vitro and in vivo by inhibiting YAP1/TAZ in endothelial cells, which increases HIF-1α expression. Our study suggests that metformin has the potential to be a therapeutic agent for fractures.

## Materials and methods

### Animals and treatments

The experimental protocols were reviewed and approved by the Ethics Committee of Xiangya Hospital of Central South University (No. 202008009). All C57BL/6 wild-type mice were purchased from Hunan SJA Laboratory Animal Co., Ltd. (Changsha, China). Mice were housed in groups of five per cage and subjected to a 12 h light/dark cycle at room temperature (R.T.) maintained at 23 °C.

For determination of the effects of metformin on normal fracture healing, 8-week-old male mice were generally anesthetized by intraperitoneal administration of sodium pentobarbital (50 mg·kg^−1^) and then subjected to unilateral stable closed femoral fracture surgery as described previously with modifications.^13^ Briefly, the right leg was depilated and sterilized, a 5 mm longitudinal incision in the skin and underlying soft tissues was made right anterolateral to the patella, and then, the patella was laterally dislocated to expose the joint space and distal femur. A 23-gage syringe needle was longitudinally inserted into the patellofemoral groove to pass through the medullary canal, and the muscles were separated by blunt dissection to expose the middle shaft femur. Then, a transverse diaphyseal fracture was generated with fine scissors. The tail of the needle was trimmed carefully and buried beneath the femoral condyle to protect the patellofemoral joint movement. After we confirmed that the fracture was not partial or comminuted, the subcutaneous tissue and skin were closed with 4–0 silk sutures. Buprenorphine was administered for pain relief. Those mice were then divided randomly into two groups: (1) the fracture + metformin group: the mice were treated intragastrically with metformin (MCE, HY-17471A, 150 mg·kg^−1^); (2) the control group: the mice were treated with an equal volume of PBS. The treatment was conducted daily with the first gavage on the same day of surgery. Samples were harvested for analysis at 2, 4, and 6 weeks after the operation.

Ten-week-old female mice were subjected to bilateral OVX surgery as described previously,^11^ followed by unilateral fracture surgery as described above two months after the OVX procedure. For evaluation of the effects of metformin on fragility fracture healing, the OVX mice were divided randomly into four groups: (1) the osteoporotic fracture + metformin group: the mice were treated intragastrically with metformin (150 mg/kg) daily; (2) the control group: the mice were treated intragastrically with an equal volume of PBS daily; (3) the osteoporotic fracture + PTH group: the mice were injected subcutaneously with PTH (ProSpec, HOR-290, 20 μg·kg^−1^) five times a week; (4) the osteoporotic fracture + ALN group: the mice were treated intragastrically with ALN (Sigma-Aldrich, A4978, 35 mg·kg^−1^) twice a week. Samples were harvested for analysis at 3, 6, and 9 weeks after fracture operation.

For analysis of the role of YAP1/TAZ in metformin-treated osteoporotic fracture healing, adeno‐associated virus serotype 2 expressing the mouse Yap1 and Taz genes (Yap1/Taz-AAV) was synthesized by Hanbio Biotechnology Co., Ltd., Shanghai and diluted in PBS to 1 × 10^12^ virus particles per mL. The construction of the Yap1/Taz-AAV and AAV control vectors is presented in Fig. S7. OVX mice were randomly divided into four groups: (1) the osteoporotic fracture + AAV control group: 10 μL of AAV control was injected locally into the fracture site bilaterally, with half of the solution injected into the soft tissue on each side of the femur; (2) the osteoporotic fracture + metformin group: the mice were treated intragastrically with metformin (150 mg·kg^−1^) daily; (3) the osteoporotic fracture + metformin + Yap1/Taz-AAV group: 10 μL each of AAV expressing Yap1 and Taz were mixed and injected locally into the fracture site, and then, the mice were treated with metformin daily; (4) the osteoporotic fracture + Yap1/Taz-AAV group. Samples were harvested for analysis 6 weeks after the fracture operation.

Whole blood samples were collected by enucleation of the eyeball after euthanasia, and serum samples were obtained by centrifugation (1 000 r·min^−1^, 10 min, R.T.). Bone marrow supernatant samples from the right tibia were obtained in heparin and centrifuged (3 000 r·min^−1^, 15 min, 4 °C). Serum and bone marrow supernatant samples were stored at −80 °C before analyses.

### Histological, immunohistochemical, and immunofluorescent analyses

For histological and immunocytochemical staining, freshly dissected femora with intact calluses were fixed in 4% paraformaldehyde for 48 h and decalcified in 0.5 mol·L^−1^ ethylenediaminetetraacetic acid (EDTA; pH = 8.0) for 10 days. After that, samples were dehydrated within graded ethanol of increasing concentration, cleared twice in xylene, and embedded in paraffin. Five micrometer-thick longitudinally oriented sections of bone were processed for hematoxylin-eosin (H&E) and Alcian blue staining. The numbers of adipocytes per square millimeter of callus area (N per mm^2^) were calculated.

For cell immunofluorescence staining, HMECs were washed with ice-cold PBS, fixed in 4% paraformaldehyde for 15 min, and then incubated in 0.1% Triton at R.T. for 15 min. After two washes with PBS, the cells were blocked with 5% fetal bovine serum (FBS) at 4 °C for 15 min. Primary antibodies against YAP1 (Cell Signaling Technology, 14074, 1:200), TAZ (Sigma-Aldrich, HPA007415, 1:200), and HIF-1α (Thermo Scientific, PA1-16601, 1:100) were incubated at 4 °C overnight, followed by secondary antibody incubation.

For bone immunofluorescence, freshly dissected femora were immediately fixed in ice-cold 4% paraformaldehyde for 4 h, followed by decalcification in 0.5 mol·L^−1^ EDTA (pH = 7.4) at 4 °C with constant shaking for 24 h. After dehydration with 20% sucrose and 2% polyvinylpyrrolidone (PVP) overnight, all samples were embedded in 8% gelatin in the presence of 20% sucrose and 2% PVP. Samples were cut into 30 μm-thick longitudinally oriented sections for immunostaining. Immunofluorescence analysis of the bone sections was performed as described previously.^9^ Briefly, after washes in PBS and permeabilization with 0.3% Triton X-100 for 30 min at R.T., the sections were blocked with 5% donkey serum at R.T. for 1 h. The primary antibodies rat anti-Emcn (Santa Cruz, sc-65495, 1:100), goat anti-CD31 (R&D Systems, FAB3628G, 1:100), rabbit anti-Ki67 (Servicebio, GB111141, 1:600), rabbit anti-YAP1 (Cell Signaling Technology, 14074, 1:100), rabbit anti-TAZ (Sigma-Aldrich, HPA007415, 1:100), rabbit anti-HIF-1α (Thermo Scientific, PA1-16601, 1:100), rabbit anti-VEGFA (Proteintech, 19003-1-AP, 1:400) and rabbit anti-LRG1 (Proteintech, 19003-1-AP, 1:400) were diluted in 0.3% Triton X-100 and incubated overnight at 4 °C. After 3 washes in PBS, the sections were incubated with species-appropriate Alexa Fluor secondary antibodies all from Jackson ImmunoResearch Laboratories diluted in 5% donkey serum for 75 min. All sections were mounted with Vectashield Antifade reagent with DAPI (Vector Laboratories, USA). Images were acquired on a Leica DMI6000B fluorescence microscope (Solms, Germany). Type H vessel area (strongly positive for both Emcn and CD31), total vessel area (positive for Emcn), Ki67^+^ endothelial cell count, and fluorescent signal intensity were quantified. Type H vessels (%) were calculated by Type H vessel area/total vessel area in the selected bone callus images.

### Microcomputed tomography (µCT) analysis

Fractured femora were dissected free of soft tissue from mice, fixed in 4% paraformaldehyde, and analyzed by high-resolution µCT (Skyscan 1176). The scanner was set at 50 kV, 400 μA, and a resolution of 11.4 μm per pixel. Image reconstruction software (NRecon v1.6), data analysis software (CTAn v1.11), and three-dimensional model visualization software (µCTVol v2.2) were applied to analyze the parameters of the calluses. The region of interest (ROI) was selected slice-by-slice, and thresholds (≥75 for the normal fracture model and ≥55 for the osteoporotic fracture model) were set to define mineralized tissue in the callus. Total callus volume (T.V.), mineralized callus volume (B.V.), and callus mineralized volume fraction (BV/TV) were quantified.

### Three-point bending test

The mechanical properties of femora were evaluated by the three-point bending test with a mechanical testing machine (Instron, 3343, USA). After removal of the intramedullary fixation pins, each femur was positioned with the posterior surface downward, with a constant vertical compression load at a speed of 5 mm per minute until rupture occurred. The ultimate stress value (N) was calculated from the load‒deformation curve.

### Enzyme-linked immunosorbent assay (ELISA)

The concentrations of OCN and VEGFA were determined using the Mouse OCN (Elabscience, E-EL-M0864c, Wuhan, China) or VEGFA (Elabscience, E-EL-M1292c) ELISA kit according to the manufacturers’ instructions. The absorbance of each well was assessed at 450 nm using a microplate reader (Hercules, Bio-Rad 680, USA) with wavelength correction set to 570 nm. The concentration of each sample was calculated according to the standard curve.

### qRT‒PCR analysis

Total RNA of cultured cells was extracted using TRIzol reagent (Thermo Scientific) following the manufacturer’s instructions. One microgram of total RNA was then reverse-transcribed into complementary DNA using All-in-One cDNA Synthesis SuperMix (Biotool, USA). qRT‒PCR was performed with FastStart Universal SYBR Premix ExTaqTM II (TaKaRa Biotechnology, Japan) on an FTC-3000 real-time PCR system (Funglyn Biotech, Canada). Relative expression was calculated for each gene by the 2^−ΔΔCT^ method, and *GAPDH* amplification was used as an internal normalization. Sequences of the primers used for each gene are listed in Supplementary Table 1.

### Cell culture

One-week-old and six-week-old mice were used for harvesting BMSCs and BMMs, respectively, by flushing the femurs and tibias as previously described.^11^ BMSCs were cultured in α-MEM (Gibco, USA) containing 10% FBS, 100 units per milliliter of penicillin, and streptomycin. Mouse embryo osteoblast precursor cells (MC3T3-E1) (Cell Bank of the Chinese Academy of Sciences, Shanghai, China) were cultured in α-MEM containing 15% FBS, 100 units per milliliter penicillin, and streptomycin. HMECs (Cell Bank of the Chinese Academy of Sciences, Shanghai, China) were cultured in MCDB131 medium (Gibco) containing 10% FBS, 1 μg·mL^−1^ hydrocortisone, 2 mmol·L^−1^ L-glutamine, and 10 ng·mL^−1^ epidermal growth factor. Cells were maintained at 37 °C in a 5% CO_2_ humidified atmosphere.

### Osteogenic and adipogenic induction of BMSCs

BMSCs were seeded and cultured in 48-well plates. When cells reached 100% confluence, the medium was replaced with osteogenic or adipogenic medium (Cyagen Biosciences, MUBMD-90021 or MUBMD-90031) with different concentrations of metformin (50, 250, 500 μmol·L^−1^) or equal volumes of PBS. BMSCs cultured in α-MEM with 10% FBS were used as a negative control. Half of the medium was replaced every other day. The cells were stained with Alizarin Red S (ARS) (Solarbio, G1452, Beijing, China) at 9 days of osteogenic differentiation or Oil Red O (ORO) (Solarbio, G1262) at 15 days of adipogenic differentiation. The percentages of ARS-positive (ARS^+^) and ORO^+^ areas were quantified with Image-Pro Plus 6 software.

### Cell proliferation assay

HMECs were seeded at a density of 5 × 10^3^ per well in 96-well culture plates, and a group without cells served as the blank. Cells were treated with metformin at various concentrations or PBS under normoxia (21% O_2_) or hypoxia (1% O_2_). After 48 h, the culture media were supplemented with CCK-8 solution (10 μL per well, Dojindo, Japan). After incubation at 37 °C for 3 h, the absorbance of each well at 450 nm was measured by using a microplate reader, and proliferation was calculated as the mean absorbance of each well minus the blank value.

### Transwell migration assay

To exclude the influence of cell proliferation on migration, we first treated HMECs with 10 μg·mL^−1^ cytochalasin C for 2 h. HMECs (1 × 10^4^ per well) were then suspended in low serum (5% FBS) medium and plated into the upper chamber of transwell 24-well plates (Corning, USA) with 8 μm pore filters under normoxic (21% O_2_) or hypoxic (1% O_2_) conditions. Five hundred microliters of complete medium (containing 10% FBS) with metformin at various concentrations or an equal volume of PBS was added to the lower chamber. After incubation for 12 h, the cells attached to the upper surface of the filter membranes were removed, and the migrated cells on the bottom side of the filter were stained with 0.5% crystal violet.

We also cocultured HMECs with BMSCs, MC3T3-E1 cells and BMMs separately to determine whether metformin could indirectly affect the function of ECs under hypoxic conditions. After treatment with 10 μg·mL^−1^ cytochalasin C for 2 h, HMECs (1 × 10^4^ per well) were plated into the upper chamber. BMSCs, MC3T3-E1 cells and BMMs (1 × 10^5^ per well) were plated into the lower chamber and supplemented with or without 50 μmol·L^−1^ metformin. After incubation for 16 h, the cells attached to the upper surface of the filter membranes were removed, and the migrated cells on the bottom side of the filter were stained with 0.5% crystal violet. The number of migrated cells was photographed with an optical microscope (Olympus, CX31, Germany) and quantified.

### Tube formation assay

HMECs were seeded at a density of 1 × 10^4^ cells per well onto 96-well plates coated with Matrigel (BD Biosciences, New Jersey, USA) and treated with metformin at various concentrations or an equal volume of PBS under normoxic (21% O_2_) or hypoxic (1% O_2_) conditions. After incubation at 37 °C for 6 h, tube formation was observed by an inverted microscope (Leica, Germany). The total branching points, total tube length, and total loops were counted by Image-Pro Plus 6 software.

### RNA interference

siRNAs targeting *YAP1* (si-*YAP1* #1, 2, and 3), *TAZ* (si-*TAZ* #1, 2, and 3), and *HIF-1α* (si-*HIF-1α* #1, 2, and 3) were obtained from RiboBio (Guangzhou, China). Cell transfection was performed according to the manufacturer’s instructions. In brief, HMECs were transfected with si-*YAP1*, si-*TAZ*, si-*HIF-1α*, or universal negative control siRNAs (si-Con) using Lipofectamine 2000 (Invitrogen, Carlsbad, CA). After incubation for 48 h, the inhibitory efficiency of these siRNAs was verified by qRT‒PCR analysis. The most effective siRNAs were used for further experiments. The target sequences of siRNAs are listed in Supplementary Table 2.

### Statistical analysis

Statistical analyses were performed using GraphPad Prism 8 software. The results are presented as the mean ± standard error of the mean (SEM) unless otherwise noted. For comparisons between two groups, we used unpaired two-tailed Student’s *t* tests. For comparisons among multiple groups, we used one-way or two-way analysis of variance (ANOVA) with the Bonferroni post hoc test. *P* values < 0.05 were considered significant. The sample size (n) for each statistical analysis is detailed in each figure legend.

### Supplementary information

Revised supplemental information-marked

Supplementary data--original images of coimmunostaining
