# Supplementary material for: Metformin accelerates bone fracture healing by promoting type H vessel formation through inhibition of YAP1/TAZ expression
Source: Bone Res. 2023 Aug 16;11:45. doi: 10.1038/s41413-023-00279-4 (PMC10432554; doi:10.1038/s41413-023-00279-4)
Supplement: Supplementary file 1 — Revised supplemental information-marked [file 41413_2023_279_MOESM1_ESM.pdf]

Supplemental information

Metformin accelerates bone fracture healing by promoting type H vessels formation through inhibited YAP1/TAZ expression

Zhe Ruan[1-2#], Hao Yin[1-4#], Teng-Fei Wan[1-4], Zhi-Rou Lin[6], Shu-Shan Zhao[1], Hai-Tao Long[1], Cheng Long[1], Zhao-Hui Li[1], Yu-Qi Liu[1], Hao Luo[1], Liang Cheng[1], Can Chen[1], Min Zeng[1], Zhang-Yuan Lin[1], Rui-Bo Zhao[1], Chun-Yuan Chen[1-5], Zhen-Xing Wang[1-5], Zheng-Zhao Liu[1-5], Jia Cao[1-5], Yi-Yi Wang[1-4], Ling Jin[1-4], Yi-Wei Liu[1-4], Guo-Qiang Zhu[1-4], Jing-Tao Zou[1-4], Jiang-Shan Gong[1-4], Yi Luo[1-4], Yin Hu[6], Yong Zhu[1*], Hui Xie[1-5*].

* Corresponding authors: Hui Xie (huixie@csu.edu.cn); Yong Zhu (doczhu2003@aliyun.com).

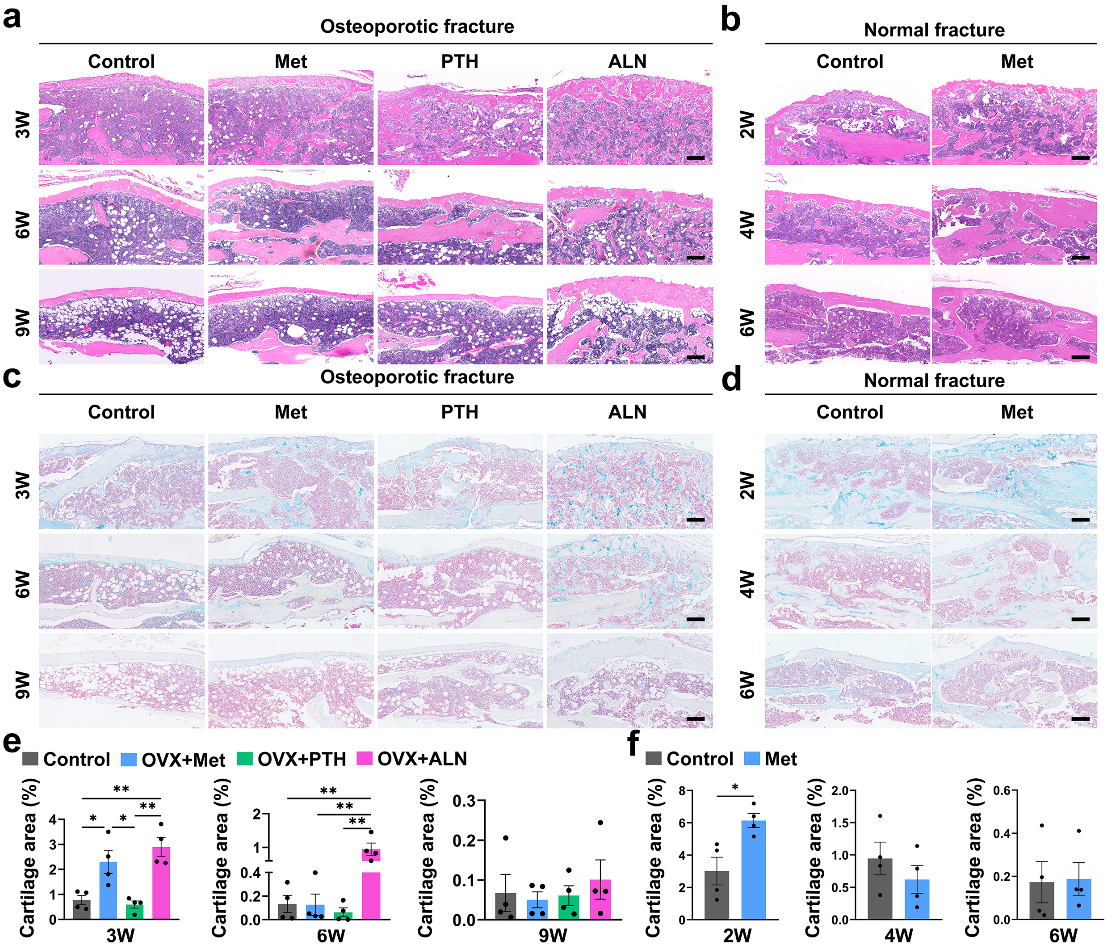

Figure. S1 Metformin enhances osteogenesis during osteoporotic and normal fracture healing.

(a-b) Representative images of H&E stained calluses from osteoporotic mice at 3, 6, and 9 weeks post-fracture **(a)** and from normal mice at 2, 4, and 6 weeks post-fracture **(b)**. Scale bar: 200 μm; Met: metformin. ALN: alendronate. PTH: parathyroid hormone. **(c-d)** Representative images of alcian blue stained calluses from osteoporotic mice at 3, 6, and 9 weeks post-fracture **(c)** and from normal mice at 2, 4, and 6 weeks post-fracture **(d)**. Scale bar: 200 μm; **(e-f)** Quantification of the cartilage area in calluses from osteoporotic **(e)** and normal **(f)** mice during fracture healing. n = 4 per group. Data were presented as mean ± SEM. $^{*}P < 0.05$, $^{**}P < 0.01$, and $^{***}P < 0.001$.

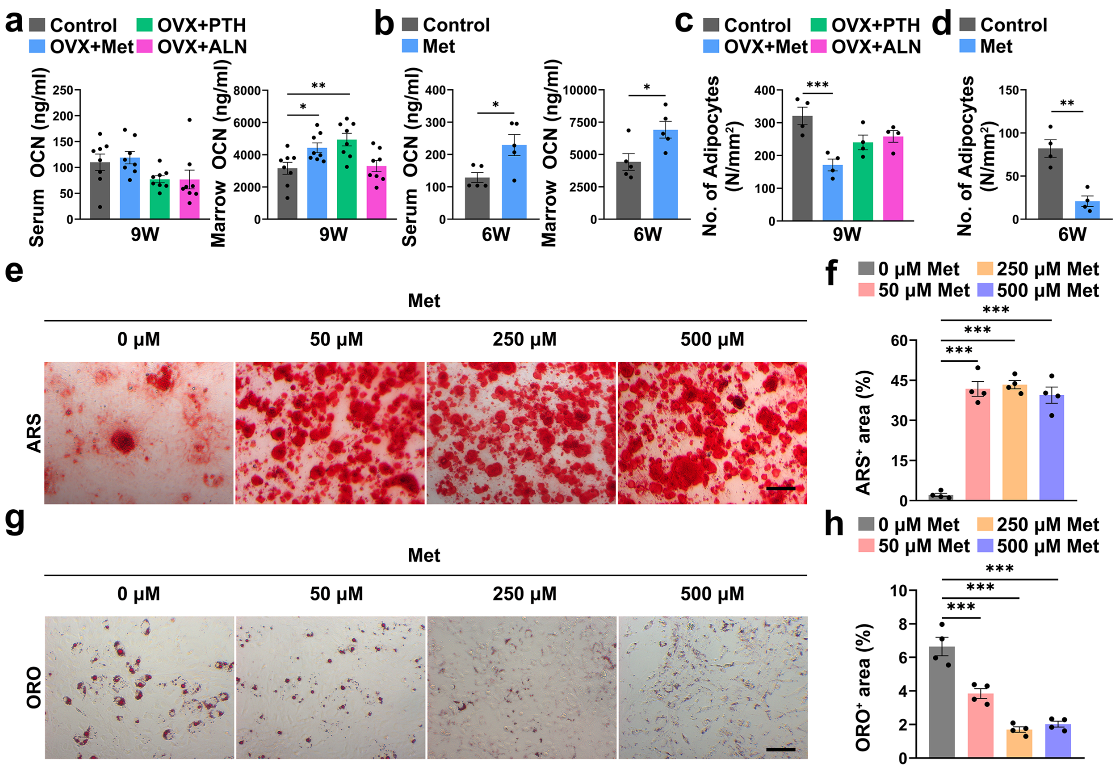

Figure. S2 Metformin enhances osteogenesis and inhibits adipogenesis *in vivo* and *in vitro*.

(a-b) ELISA for the serum and bone marrow OCN concentrations at the endpoint of osteoporotic **(a)** (n = 8 per group) and normal **(b)** fracture healing (n = 5 per group). **(c-d)** Quantification of the number of adipocytes in calluses at the endpoint of osteoporotic **(c)** and normal **(d)** fracture healing. n = 4 per group. **(e-f)** Representative Alizarin Red S staining images **(e)** of mineralized nodules of BMSCs treated with metformin in different concentrations after osteogenic induction for 9 days and quantitation of ARS positive areas per field **(f)**. Scale bar: 100 μm. n = 4 per group; **(g-h)** Representative Oil Red O staining images **(g)** of lipid droplets of BMSCs treated with metformin in different concentrations after adipogenic induction for 15 days and quantitation of ORO positive areas per field **(h)**. Scale bar: 100 μm. n = 4 per group; Data were presented as mean ± SEM. $^{*}P < 0.05$, $^{**}P < 0.01$, and $^{***}P < 0.001$.

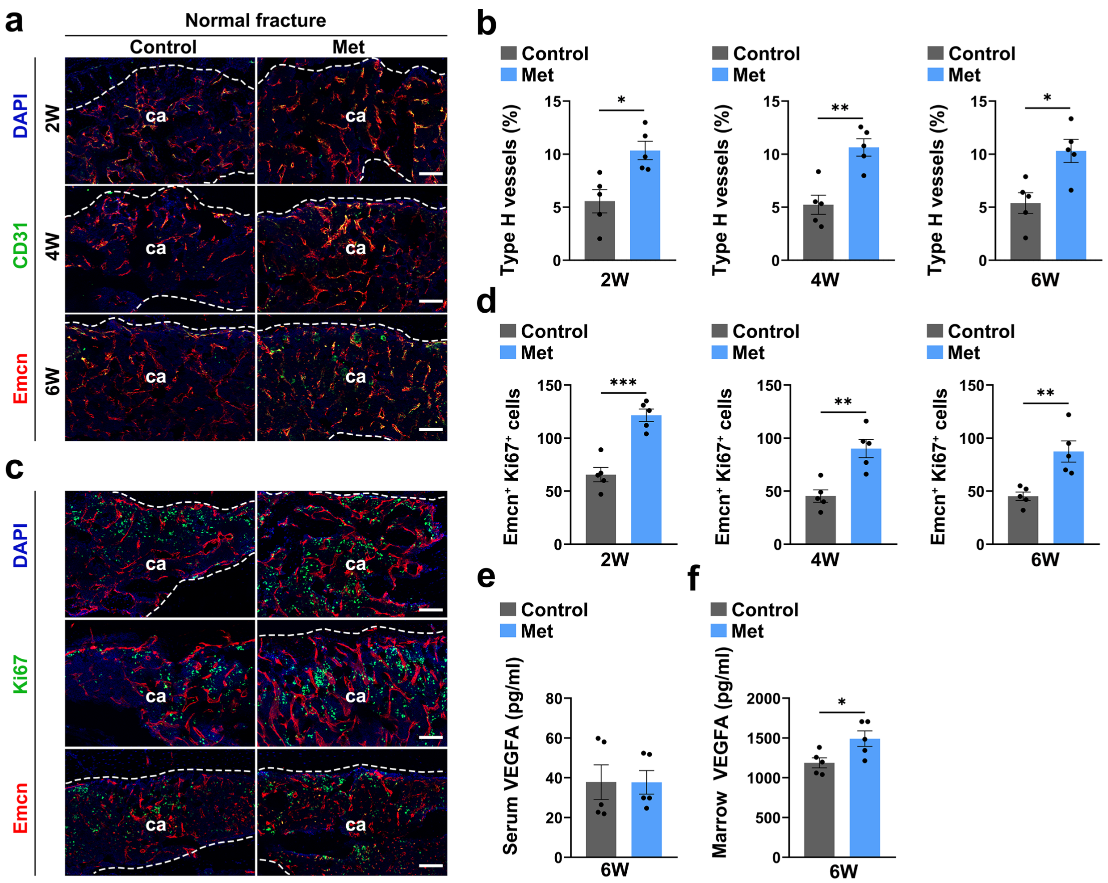

Figure. S3 Metformin promotes type H vessel formation in normal fracture mice. **(a-b)** Representative CD31 and Emcn co-immunostaining images **(a)** with quantification of type H vessels ratio in calluses from normal mice treated with PBS or metformin at 2, 4, and 6 weeks post-fracture **(b)**. ca: callus. The dotted line represents the boundary of calluses. Met: metformin. Scale bar: 100 μm. n = 5 per group. **(c-d)** Representative Ki67 and Emcn co-immunostaining images **(c)** with quantification of Ki67 positive endothelial cells number in calluses from normal mice treated with PBS and Met at 2, 4, and 6 weeks post-fracture **(d)**. Scale bar: 100 μm. n = 5 per group. **(e-f)** ELISA for the serum **(e)** and bone marrow **(f)** concentrations of VEGFA at 6 weeks post-normal fracture. n = 8 per group; Data were presented as mean ± SEM. $^{*}P < 0.05$, $^{**}P < 0.01$, and $^{***}P < 0.001$.

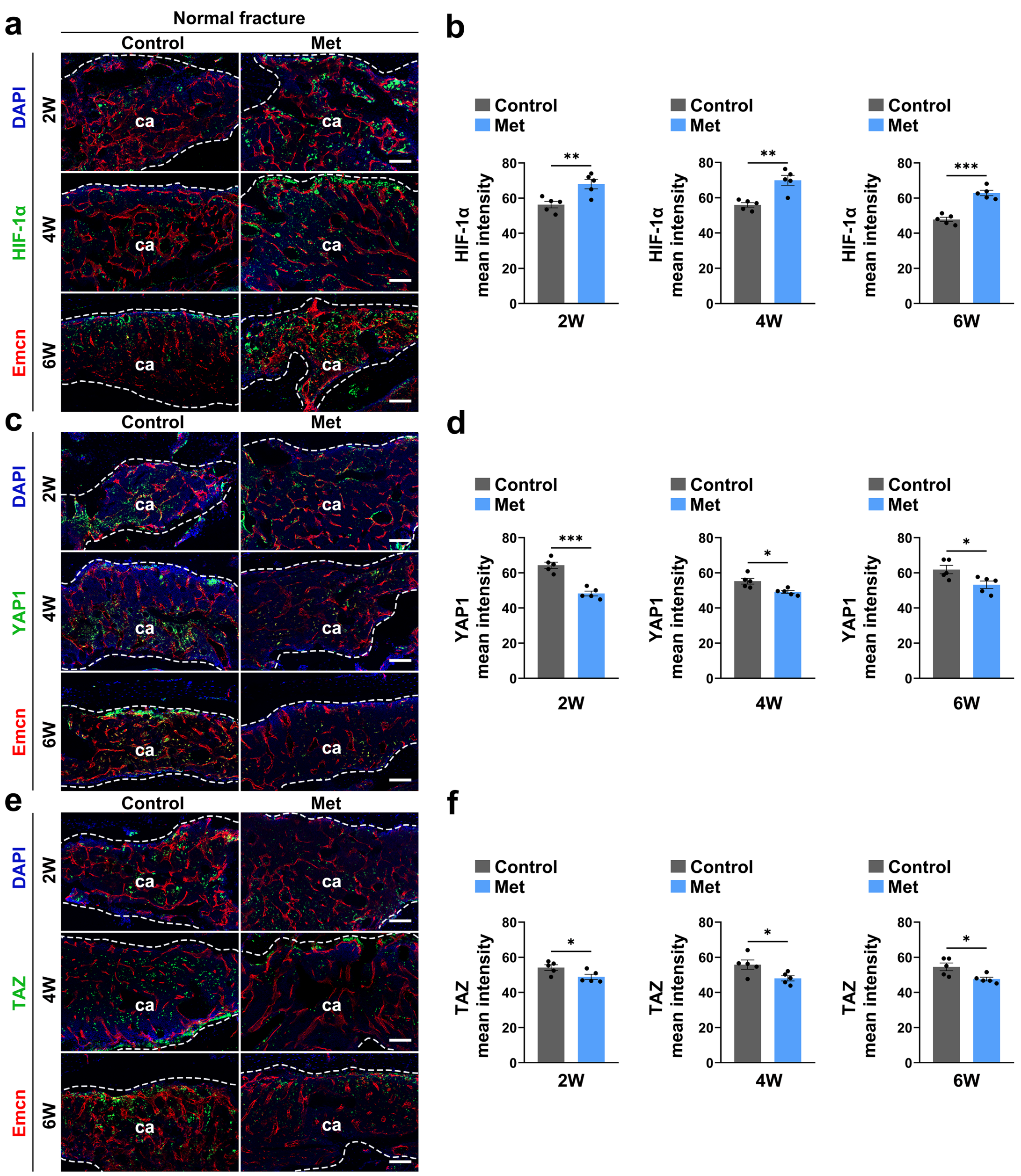

Figure. S4 Metformin promotes the expression of HIF-1α by inhibiting the expression of YAP1/TAZ during normal fracture healing

(a-b) Representative HIF-1α and Emcn co-immunostaining images **(a)** with quantification of the mean intensity of HIF-1α in calluses from normal mice treated with PBS or metformin at 2, 4, and 6 weeks post-fracture **(b)**. ca: callus. The dotted line represents the boundary of calluses. Met: metformin. Scale bar: 100 µm. n = 5 per group.

(c-d) Representative YAP1 and Emcn co-immunostaining images **(c)** with

quantification of the mean intensity of YAP1 in calluses from normal mice at 2, 4, and 6 weeks post-fracture **(d)**. Scale bar: 100 μm. n = 5 per group. **(e-f)** Representative TAZ and Emcn co-immunostaining images **(e)** with quantification of the mean intensity of TAZ in calluses from normal mice at 2, 4, and 6 weeks post-fracture **(f)**. Scale bar: 100 μm. n = 5 per group. Data were presented as mean ± SEM. $^{*}P < 0.05$, $^{**}P < 0.01$, and $^{***}P < 0.001$.

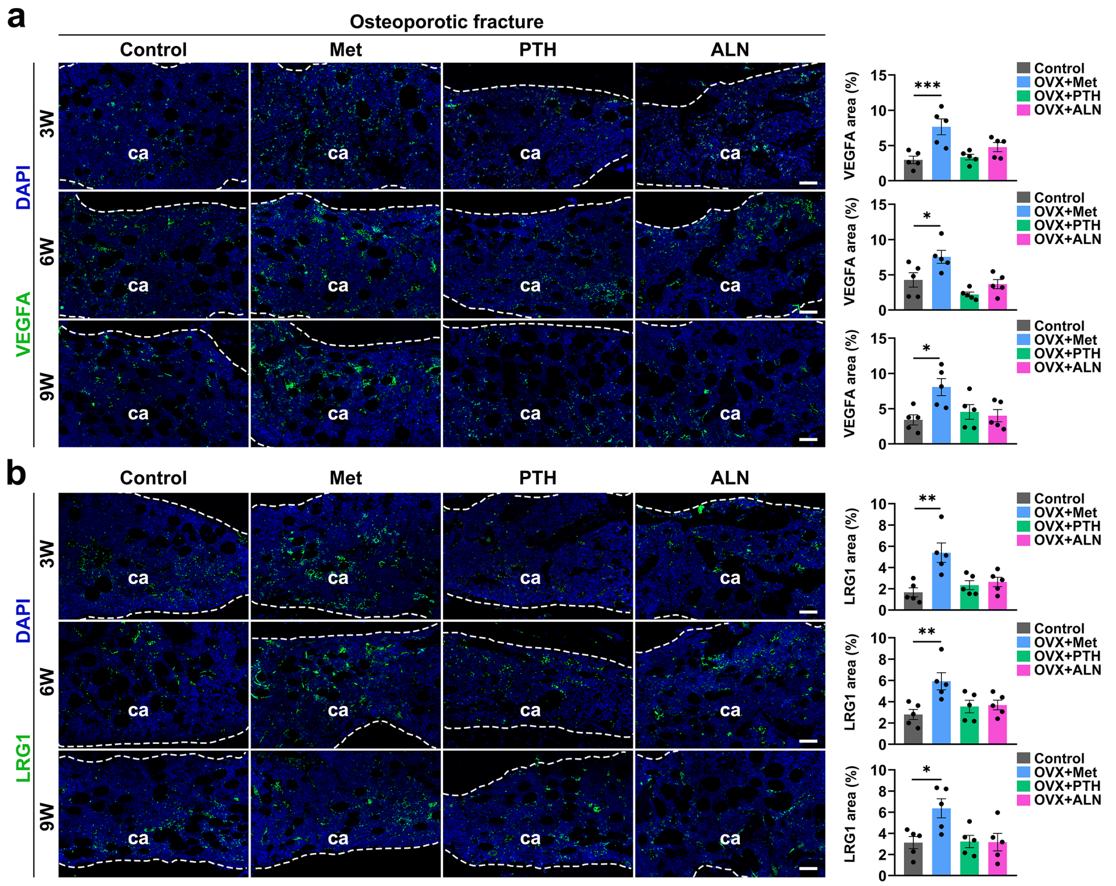

Figure. S5 Metformin promotes the expression of VEGFA and LRG1 during osteoporotic fracture healing.

(a) Representative immunostaining images (left) with quantification (right) of VEGFA in calluses from osteoporotic mice treated with PBS, Met, PTH, and ALN at 3, 6, and 9 weeks post-fracture. ca: callus. The dotted line represents the boundary of calluses. Met: metformin. ALN: alendronate. PTH: parathyroid hormone. Scale bar: 50 μm. n = 5 per group. **(b)** Representative immunostaining images (left) with quantification (right) of LRG1 in calluses from osteoporotic mice treated with PBS, Met, PTH, and ALN at 3, 6, and 9 weeks post-fracture. Scale bar: 50 μm. n = 5 per group. Data were presented as mean ± SEM. $^{*}P < 0.05$, $^{**}P < 0.01$, and $^{***}P < 0.001$.

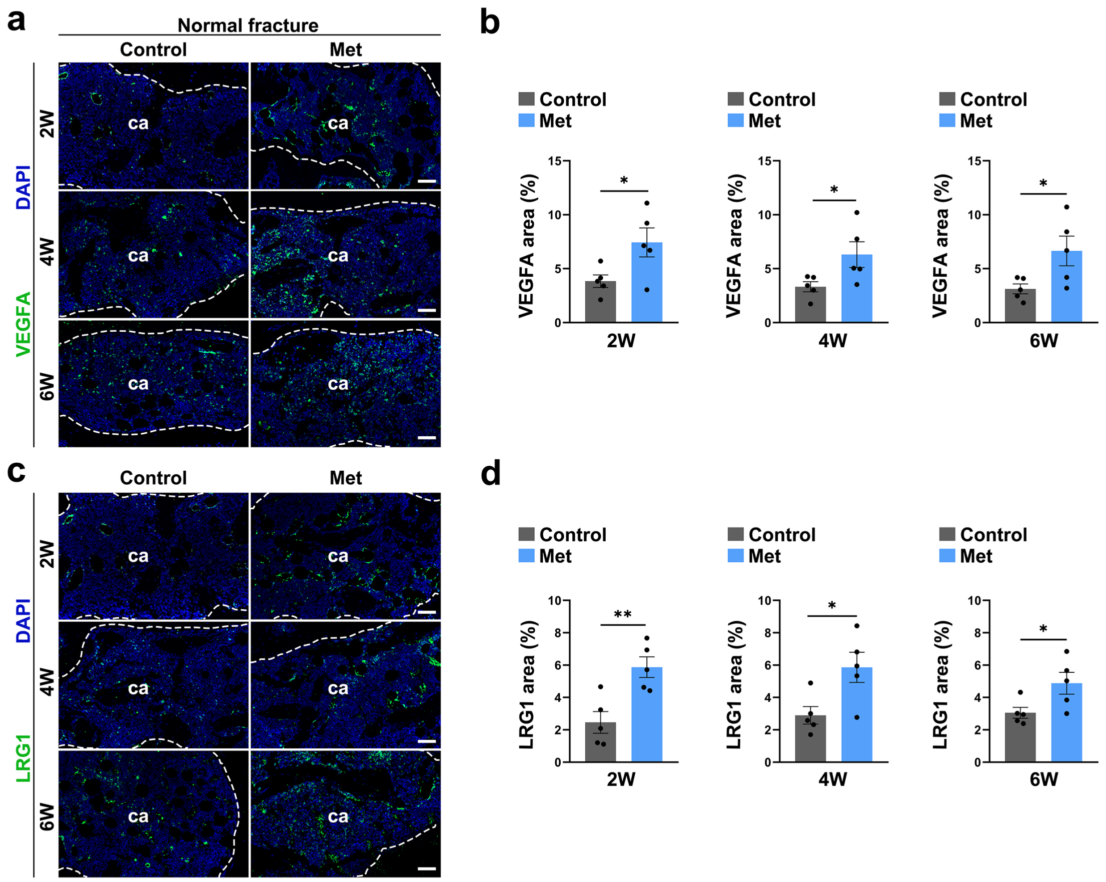

Figure. S6 Metformin promotes the expression of VEGFA and LRG1 during normal fracture healing.

(a) Representative immunostaining images (left) with quantification (right) of VEGFA in calluses from normal mice treated with PBS and Met at 2, 4, and 6 weeks post-fracture. ca: callus. The dotted line represents the boundary of calluses. Met: metformin. Scale bar: 50 μm. n = 5 per group. **(b)** Representative immunostaining images (left) with quantification (right) of LRG1 in calluses from normal mice treated with PBS and Met at 2, 4, and 6 weeks post-fracture. ca: callus. The dotted line represents the boundary of calluses. Met: metformin. Scale bar: 50 μm. n = 5 per group. Data were presented as mean ± SEM. $^{*}P < 0.05$ and $^{**}P < 0.01$.

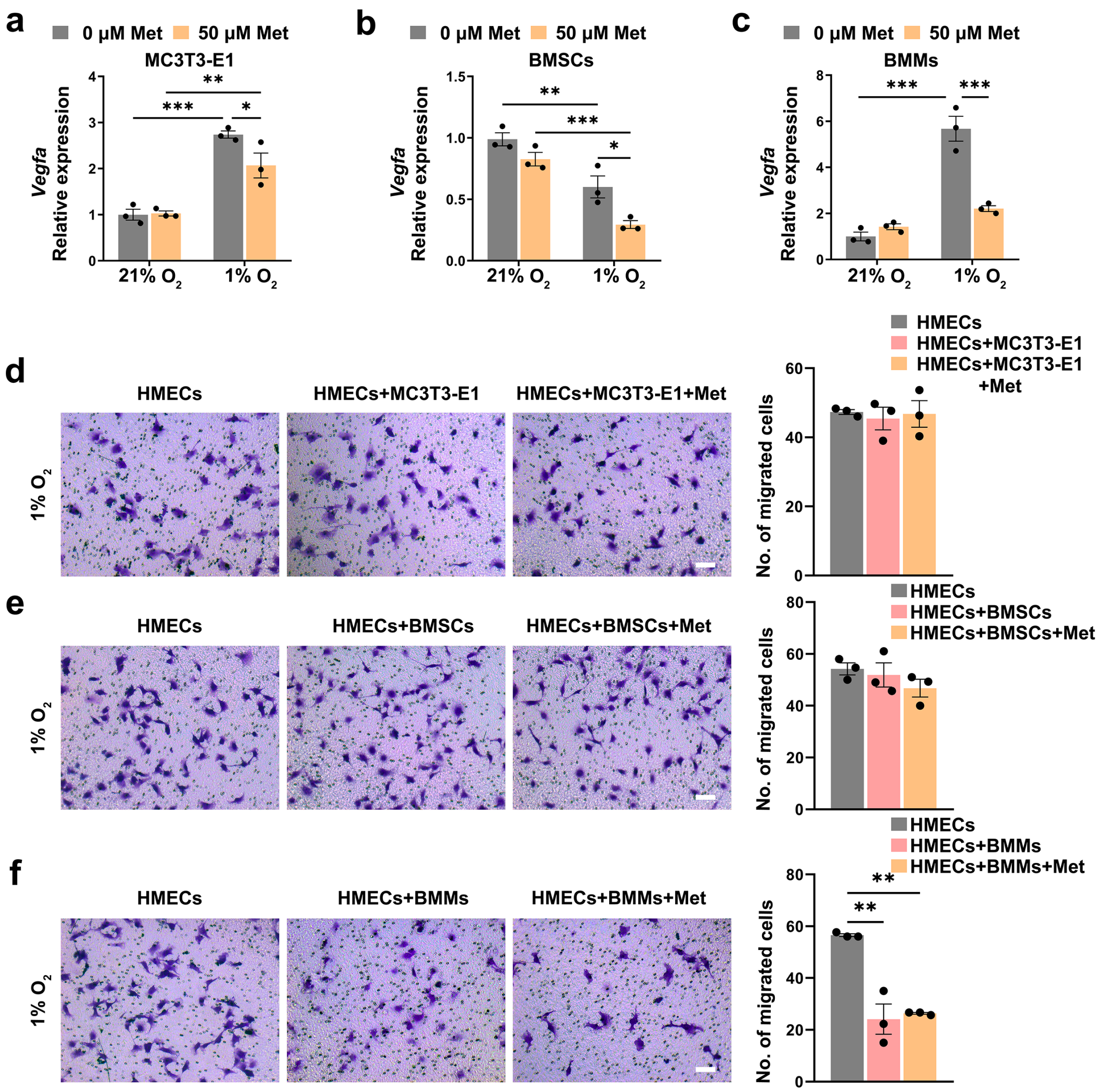

Figure. S7 Metformin enhances angiogenesis under hypoxic conditions independently of its effects on osteoblast progenitors and bone marrow-derived macrophages.

(a-c) qRT-PCR analysis for *Vegfa* mRNA level in osteoblast progenitors MC3T3-E1 cells **(a)**, BMSCs **(b)**, and BMMs **(c)** with or without metformin treatment under different oxygen concentrations. Met: metformin. n = 3 per group. **(d-f)** Representative images of transwell migration assay (left) with quantification of crystal violet-stained migrated HMECs (right) co-cultured with MC3T3-E1 cells **(d)**, BMSCs **(e)**, and BMMs **(f)** with or without metformin treatment under hypoxic conditions (1% O_2). Scale bar:

100 µm. n = 3 per group. Data were presented as mean ± SEM. $^{*}P < 0.05$, $^{**}P < 0.01$, and $^{***}P < 0.001$.

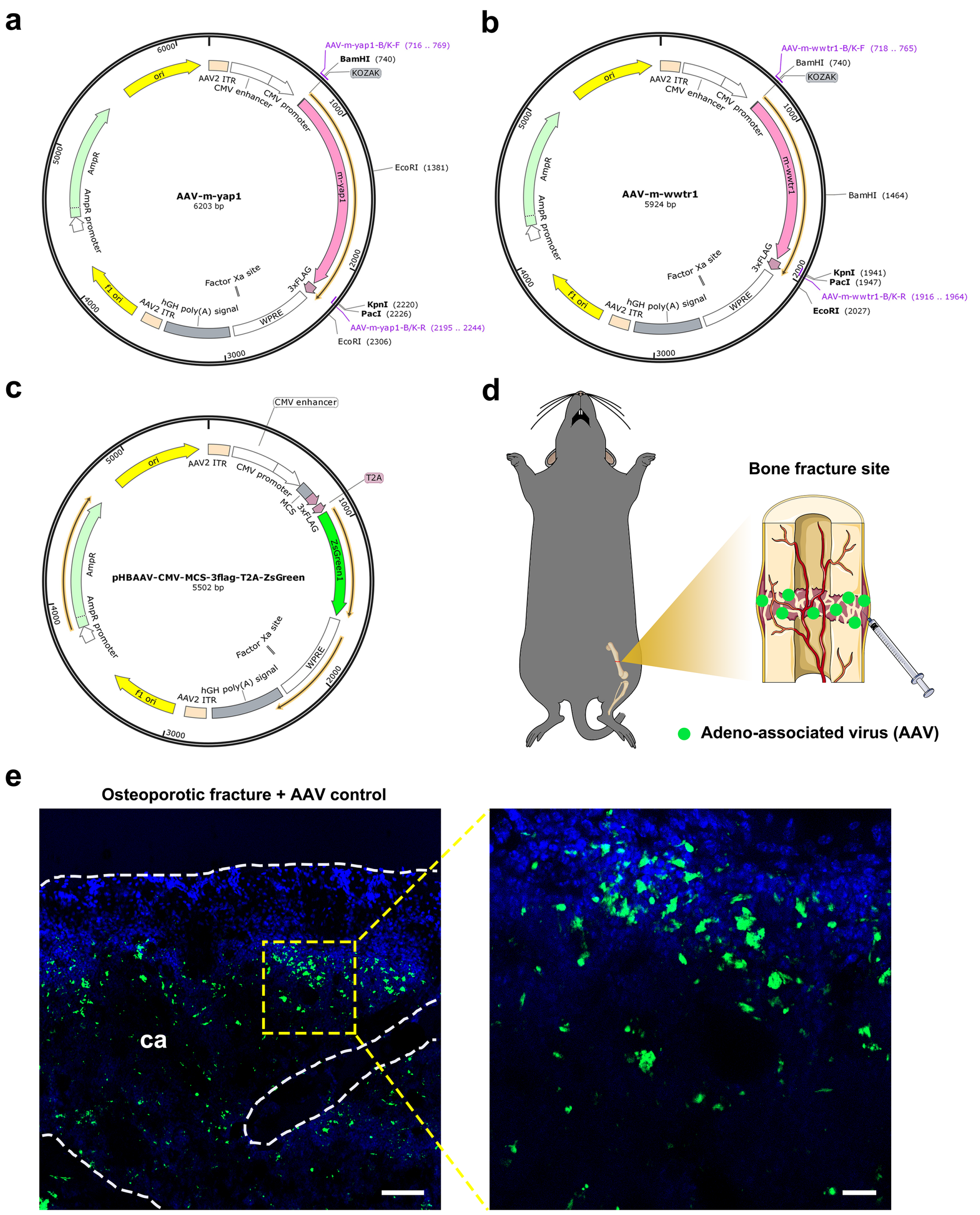

Figure. S8 AAV construction and mice model of injection of the AAV into the fracture site.

(a-c) Construction of AAV2 expressing Yap1 **(a)**, Taz **(b)**, and AAV control **(c)**. Note that Yap1/Taz-AAV itself does not express fluorescence while AAV control expresses green fluorescence (ZsGreen). **(d)** Schematic of the AAV injection into the bone

fracture area. **(e)** Immunofluorescence of ZsGreen expression in callus (left) with the magnified area (right) in the boxed area in the left image of osteoporotic mice that received AAV control injections at 6 weeks after fracture operation. The AAV control group expressed ZsGreen as a reporter of AAV2 infection efficiency. ca: callus. The dotted line represents the boundary of calluses. Scale bar, left 100 μm; right 20 μm.

Supplementary Table 1. Primer sequences for qRT-PCR.

Gene	Forward (5’-3’)	Reverse (5’-3’)
h-HIF-1α	AGAGGTTGAGGGACGGAGAT	GACGTTCAGAACTTATCCTACCAT
h-YAP1	GAACTGCTTCGGCAGGTGAG	GCAGGGCTAACTCCTGACATT
h-TAZ	TCACATCCTGGCGACTCTCA	GAGGCCGGATTCATCTTCTGG
h-VEGFA	ACATCACCATGCAGATTATGCG	CTCCAGGGCATTAGACAGCA
h-LRG1	GACAGCGACCAAAAAGCCCA	TGAAGAATTCCACGGCCAGG
h-GAPDH	GGATTTGGTCGTATTGGGCG	TCCCGTTCTCAGCCATGTAGT
m-VEGFA	GCACATAGAGAGAATGAGCTTCC	CTCCGCTCTGAACAAGGCT
m-GAPDH	AGGTCGGTGTGAACGGATTTG	TGTAGACCATGTAGTTGAGGTCA

Supplementary Table 2. siRNA target Sequences.

**siRNA**	**Target sequences**
si-*HIF-1α* #1	GGAACATGATGGTTCACTT
si-*HIF-1α* #2	CTACCCACATACATAAAGA
si-*HIF-1α* #3	CCAGCAACTTGAGGAAGTA
si-*YAP1* #1	CCACCAAGCTAGATAAAGA
si-*YAP1* #2	GAGATGGAATGAACATAGA
si-*YAP1* #3	GTAGCCAGTTACCAACACT
si-*TAZ* #1	CGATGAATCAGCCTCTGAA
si-*TAZ* #2	GGACAAACACCCATGAACA
si-*TAZ* #3	AGAGTCTGCTCTGAACAAA
