# Supplementary material for: Metformin accelerates bone fracture healing by promoting type H vessel formation through inhibition of YAP1/TAZ expression
Source: Bone Res. 2023 Aug 16;11:45. doi: 10.1038/s41413-023-00279-4 (PMC10432554; doi:10.1038/s41413-023-00279-4)
Supplement: Supplementary file 2 — Supplementary data--original images of coimmunostaining [file 41413_2023_279_MOESM2_ESM.pdf]

Figure 3

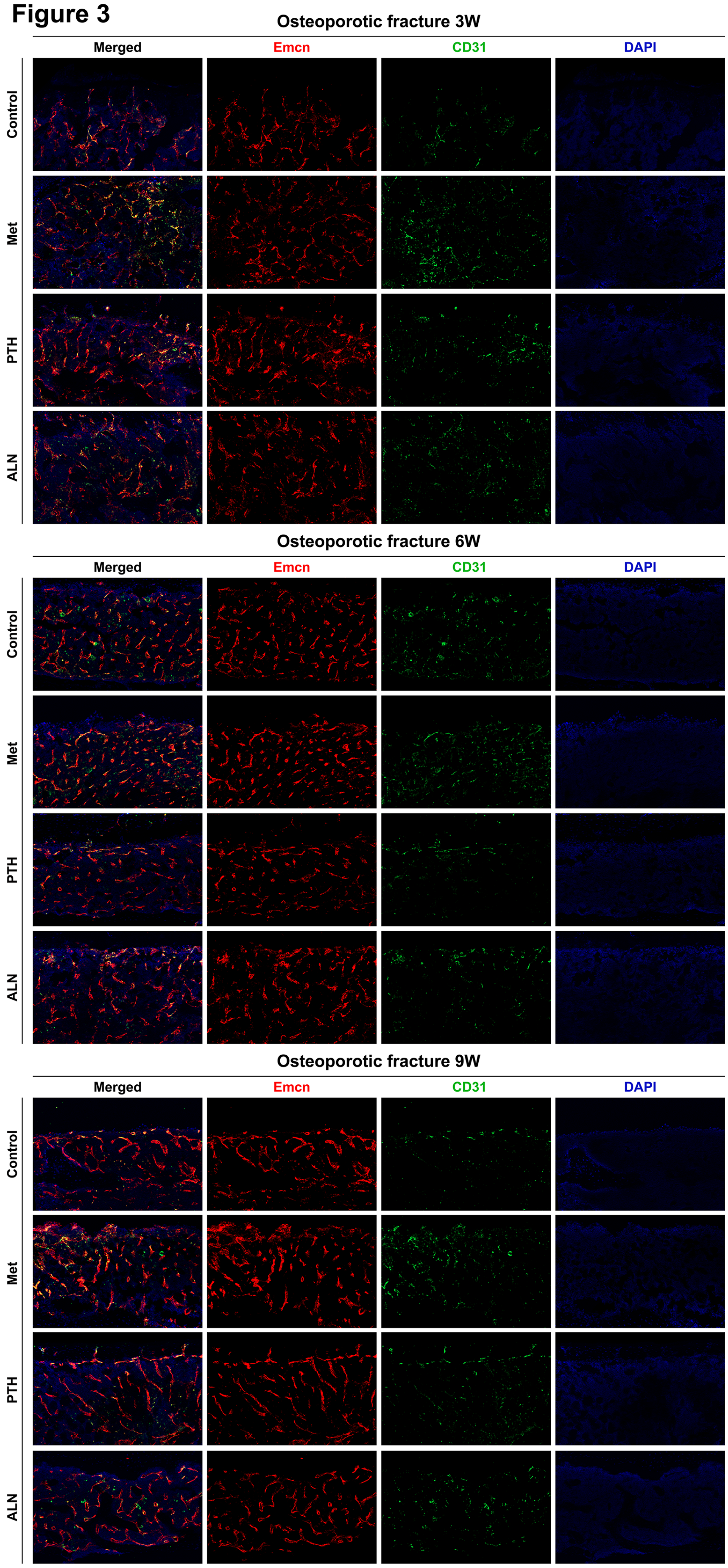
Osteoporotic fracture 3W
Merged
Emcn
CD31
DAPI
Control
Met
PTH
ALN
Osteoporotic fracture 6W
Merged
Emcn
CD31
DAPI
Control
Met
PTH
ALN
Osteoporotic fracture 9W
Merged
Emcn
CD31
DAPI
Control
Met
PTH
ALN

Figure 3

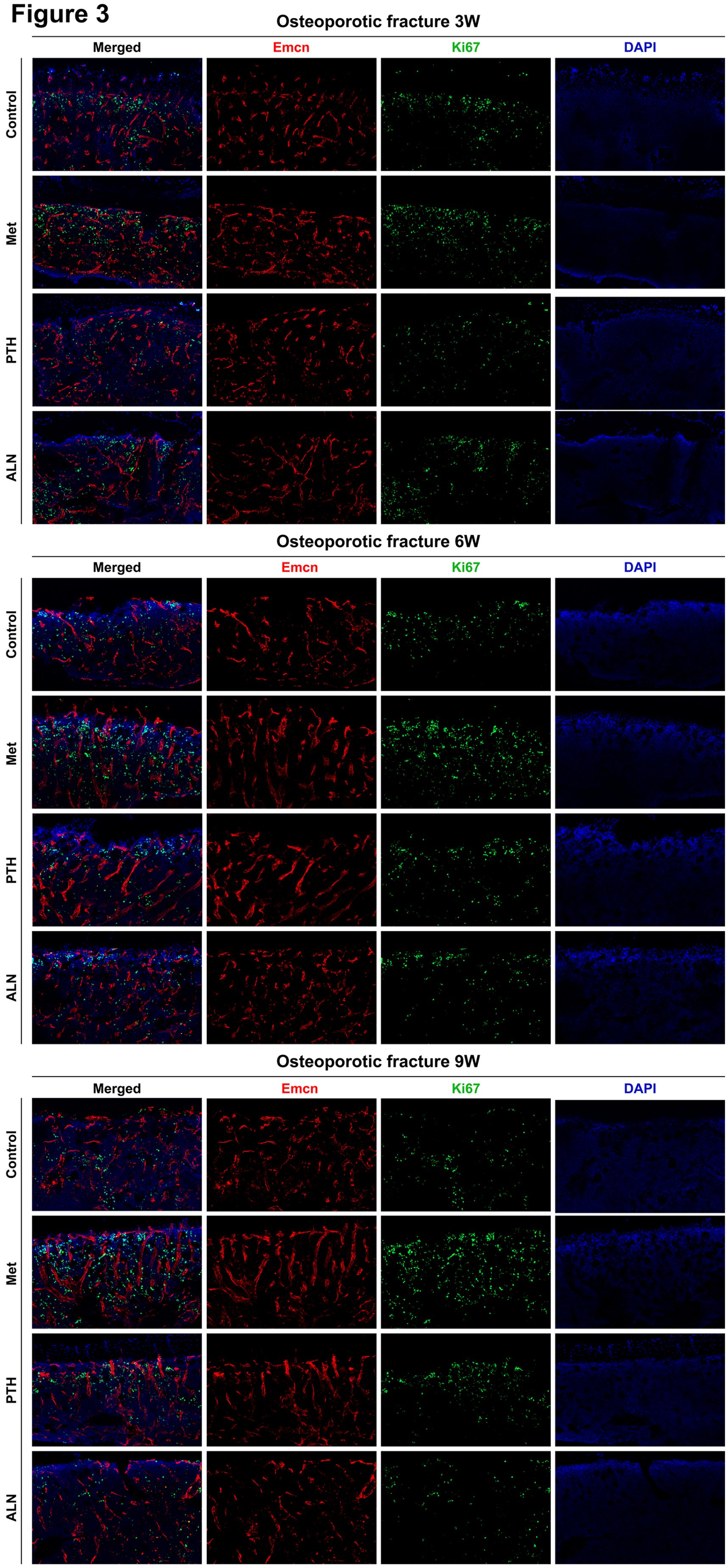
Osteoporotic fracture 3W
Merged
Emcn
Ki67
DAPI
Control
Met
PTH
ALN
Osteoporotic fracture 6W
Merged
Emcn
Ki67
DAPI
Control
Met
PTH
ALN
Osteoporotic fracture 9W
Merged
Emcn
Ki67
DAPI
Control
Met
PTH
ALN

Figure 5

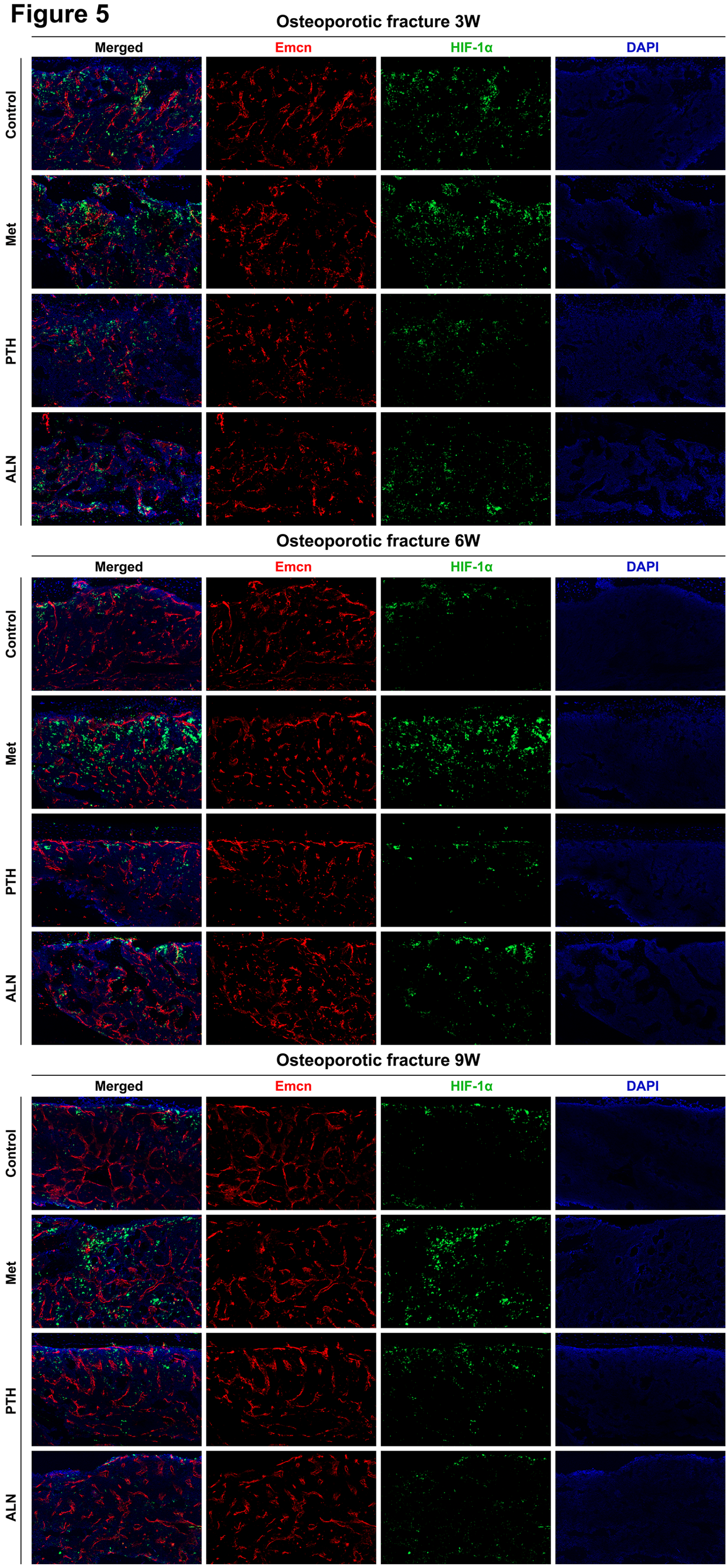

Osteoporotic fracture 3W
Merged
Emcn
HIF-1α
DAPI
Control
Met
PTH
ALN
Osteoporotic fracture 6W
Merged
Emcn
HIF-1α
DAPI
Control
Met
PTH
ALN
Osteoporotic fracture 9W
Merged
Emcn
HIF-1α
DAPI
Control
Met
PTH
ALN

Figure 5

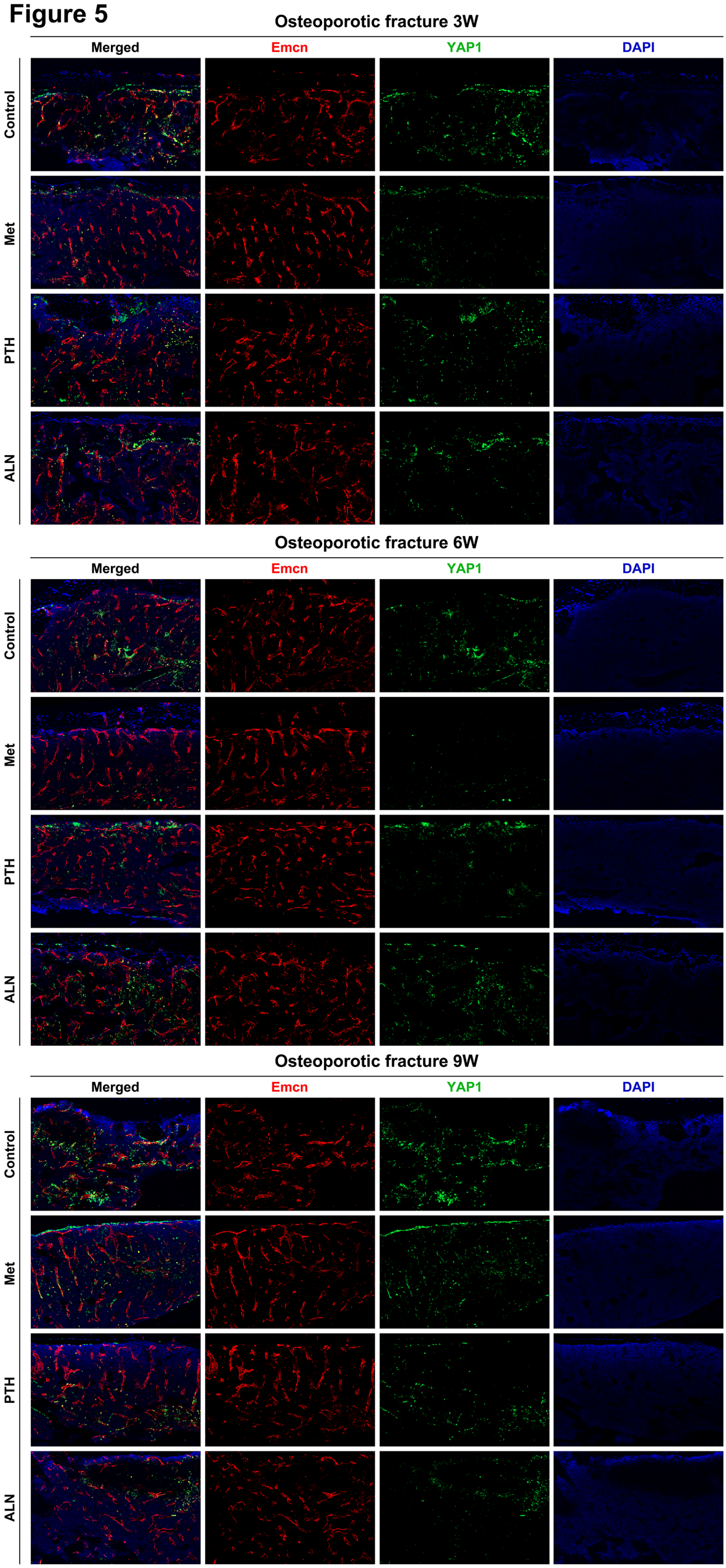
Osteoporotic fracture 3W
Merged
Emcn
YAP1
DAPI
Control
Met
PTH
ALN
Osteoporotic fracture 6W
Merged
Emcn
YAP1
DAPI
Control
Met
PTH
ALN
Osteoporotic fracture 9W
Merged
Emcn
YAP1
DAPI
Control
Met
PTH
ALN

Figure 5

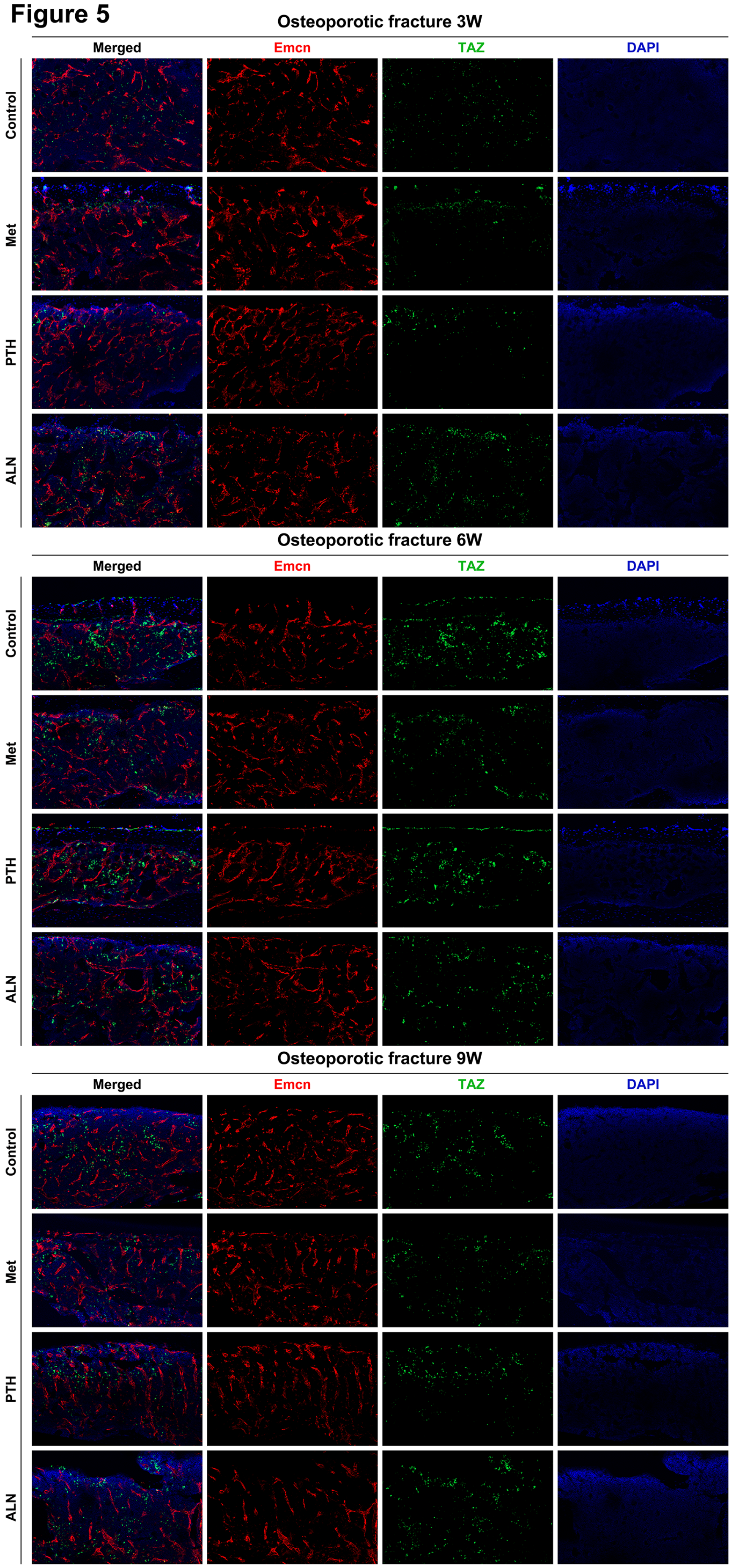
Osteoporotic fracture 3W
Merged
Emcn
TAZ
DAPI
Control
Met
PTH
ALN
Osteoporotic fracture 6W
Merged
Emcn
TAZ
DAPI
Control
Met
PTH
ALN
Osteoporotic fracture 9W
Merged
Emcn
TAZ
DAPI
Control
Met
PTH
ALN

Figure 6

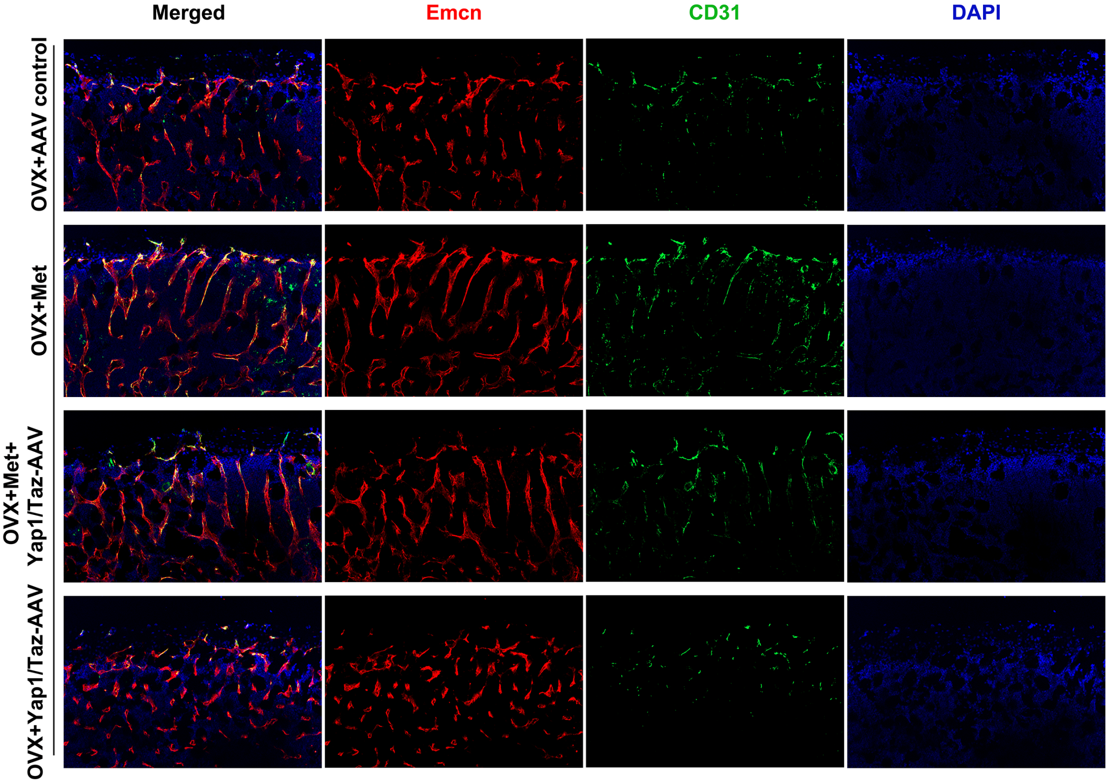

Figure 6

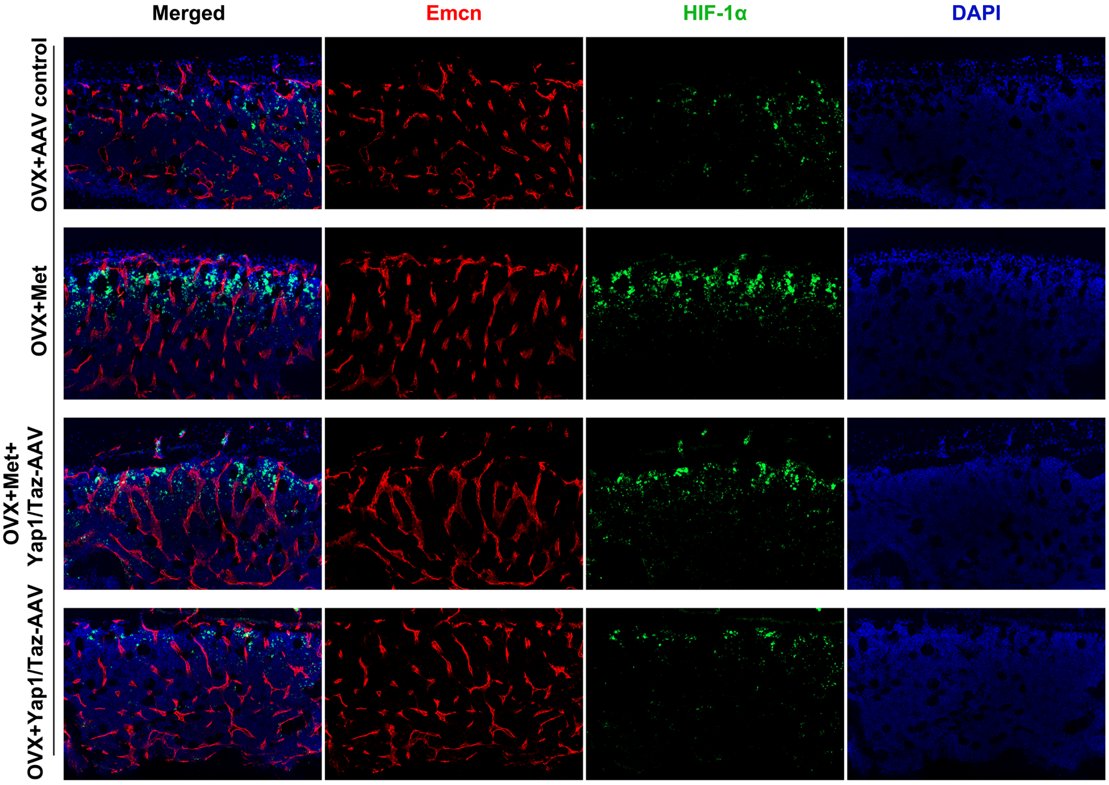

Figure 6

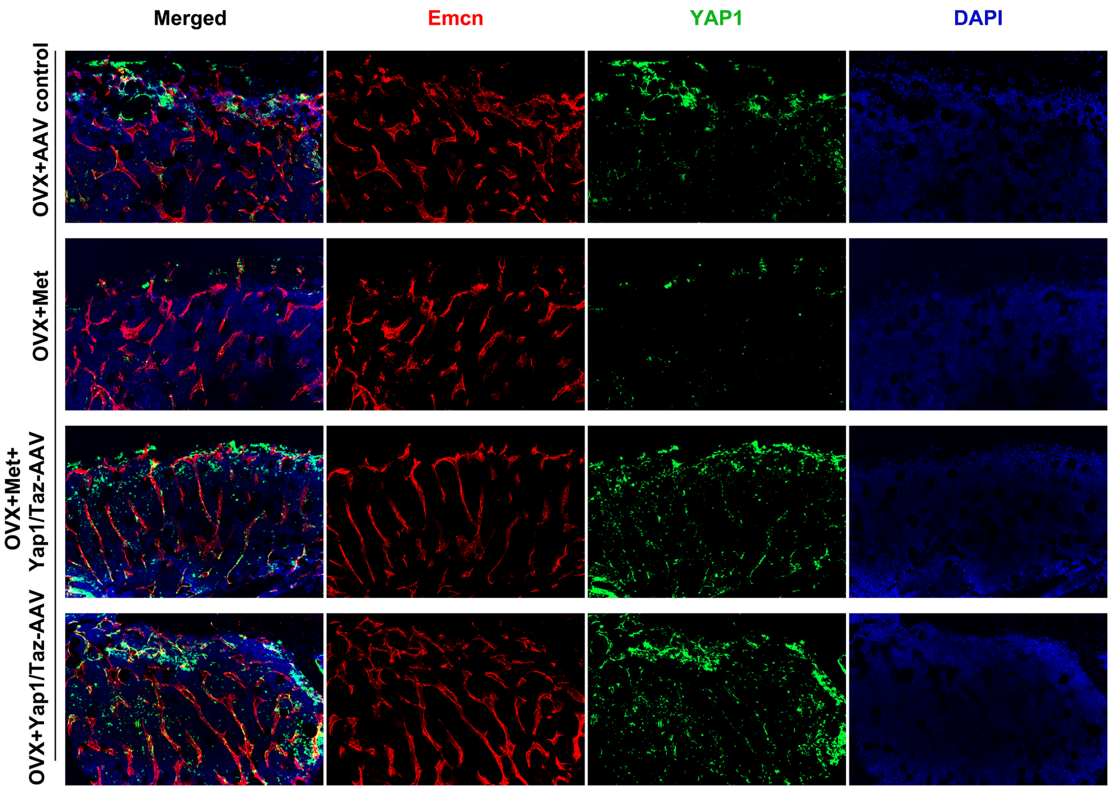

Figure 6

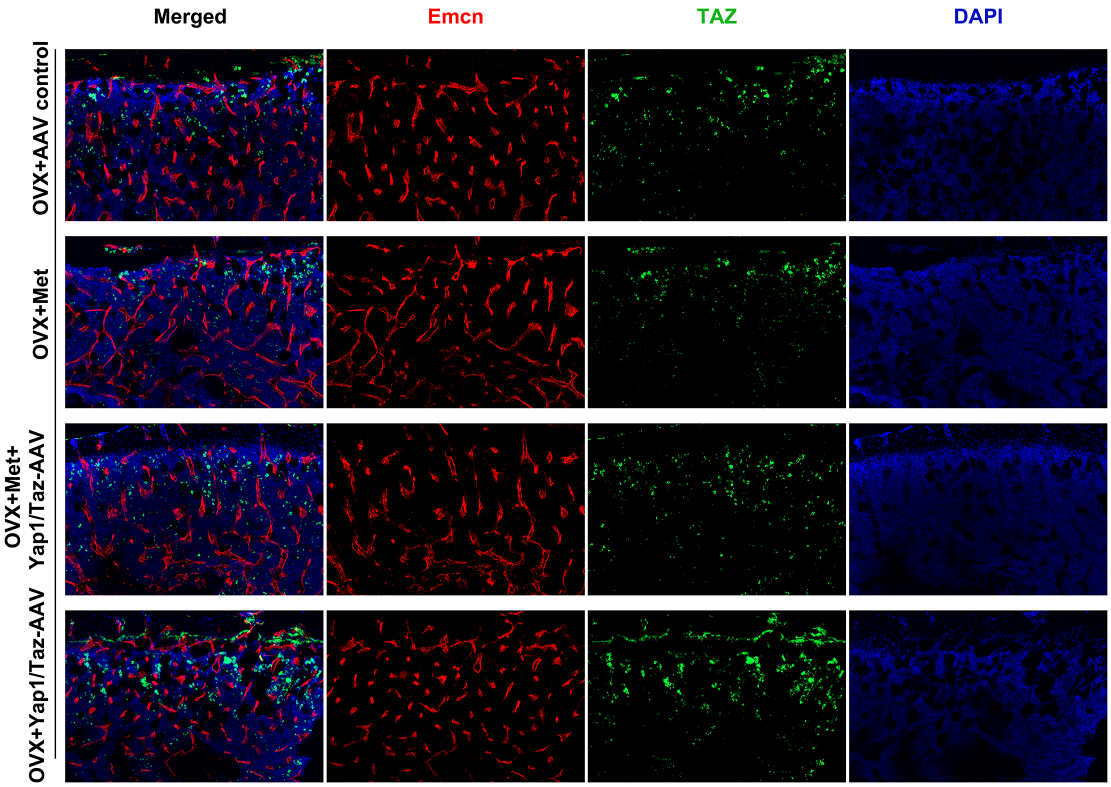

Figure 6

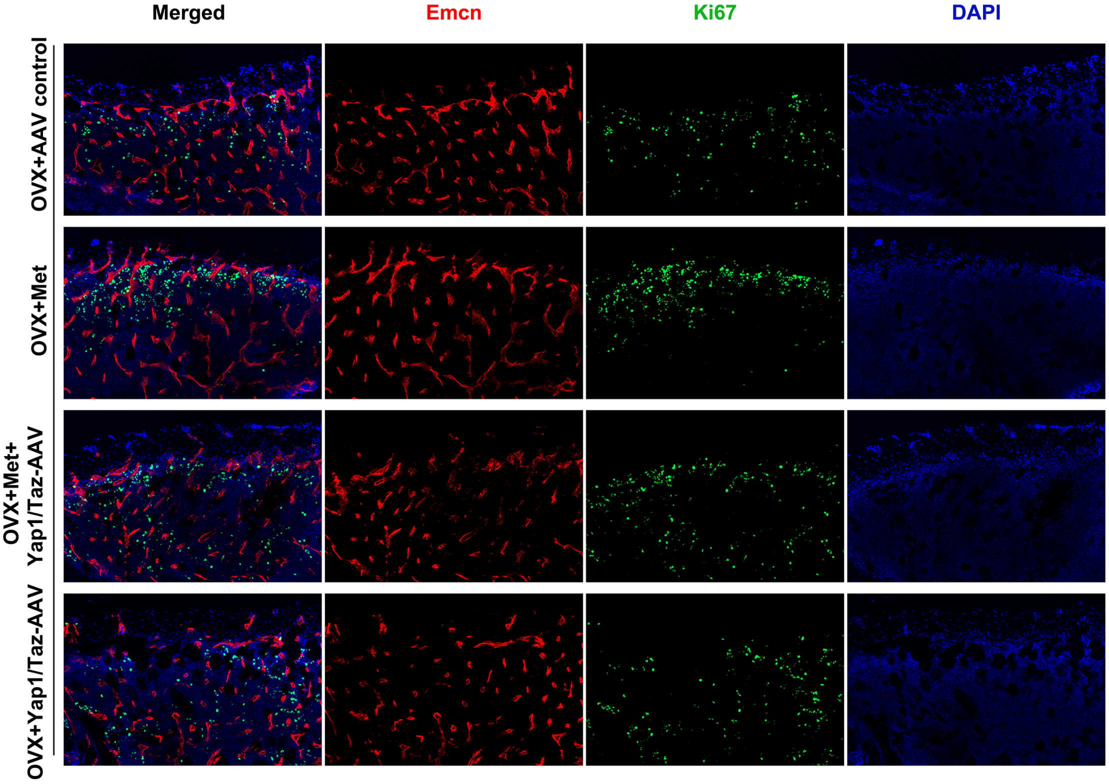

Merged
Emcn
Ki67
DAPI
OVX+AAV control
OVX+Met
OVX+Met+
Yap1/Taz-AAV
OVX+Yap1/Taz-AAV

Figure S3

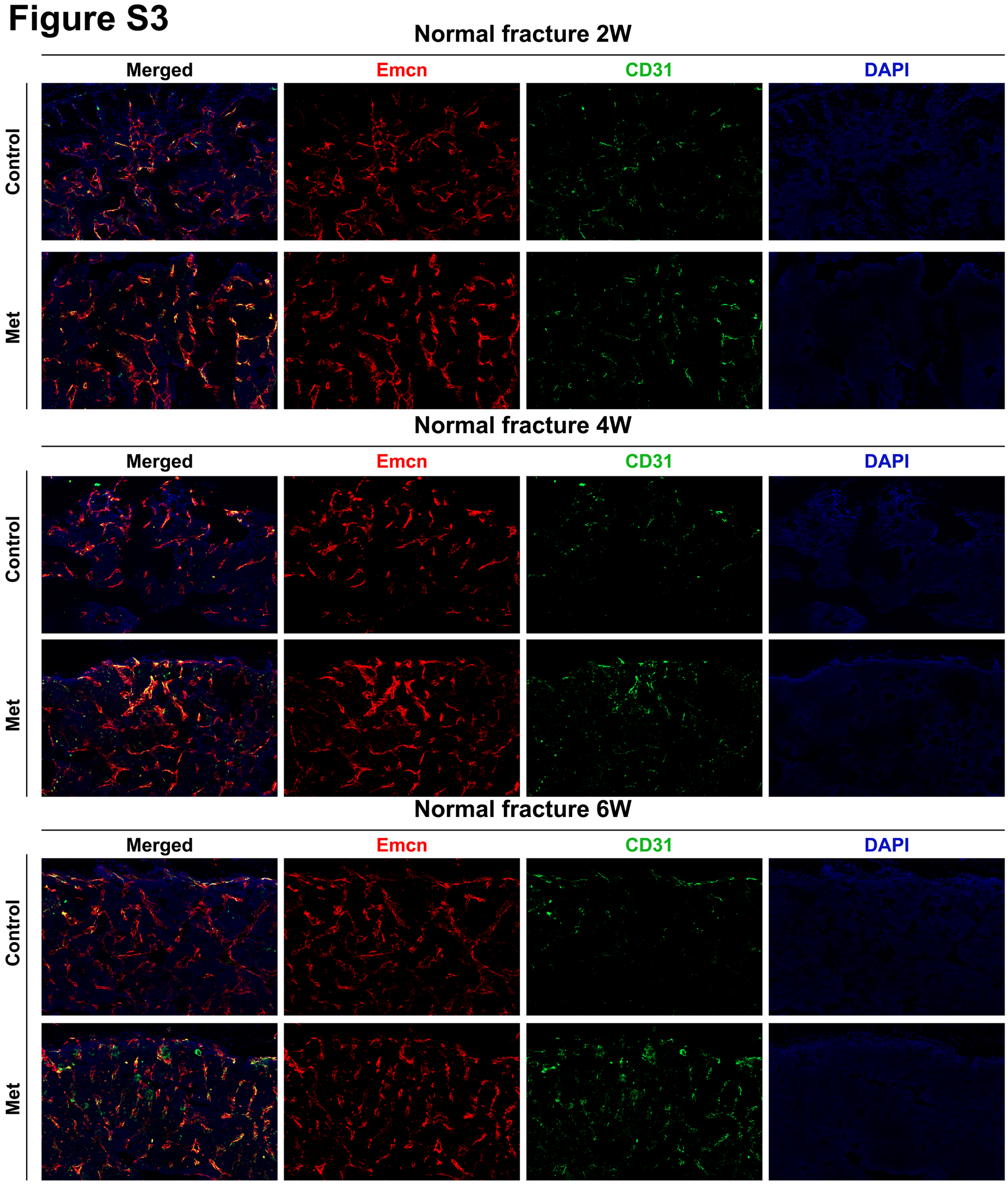
Normal fracture 2W
Merged
Emcn
CD31
DAPI
Control
Met
Normal fracture 4W
Merged
Emcn
CD31
DAPI
Control
Met
Normal fracture 6W
Merged
Emcn
CD31
DAPI
Control
Met

Figure S3

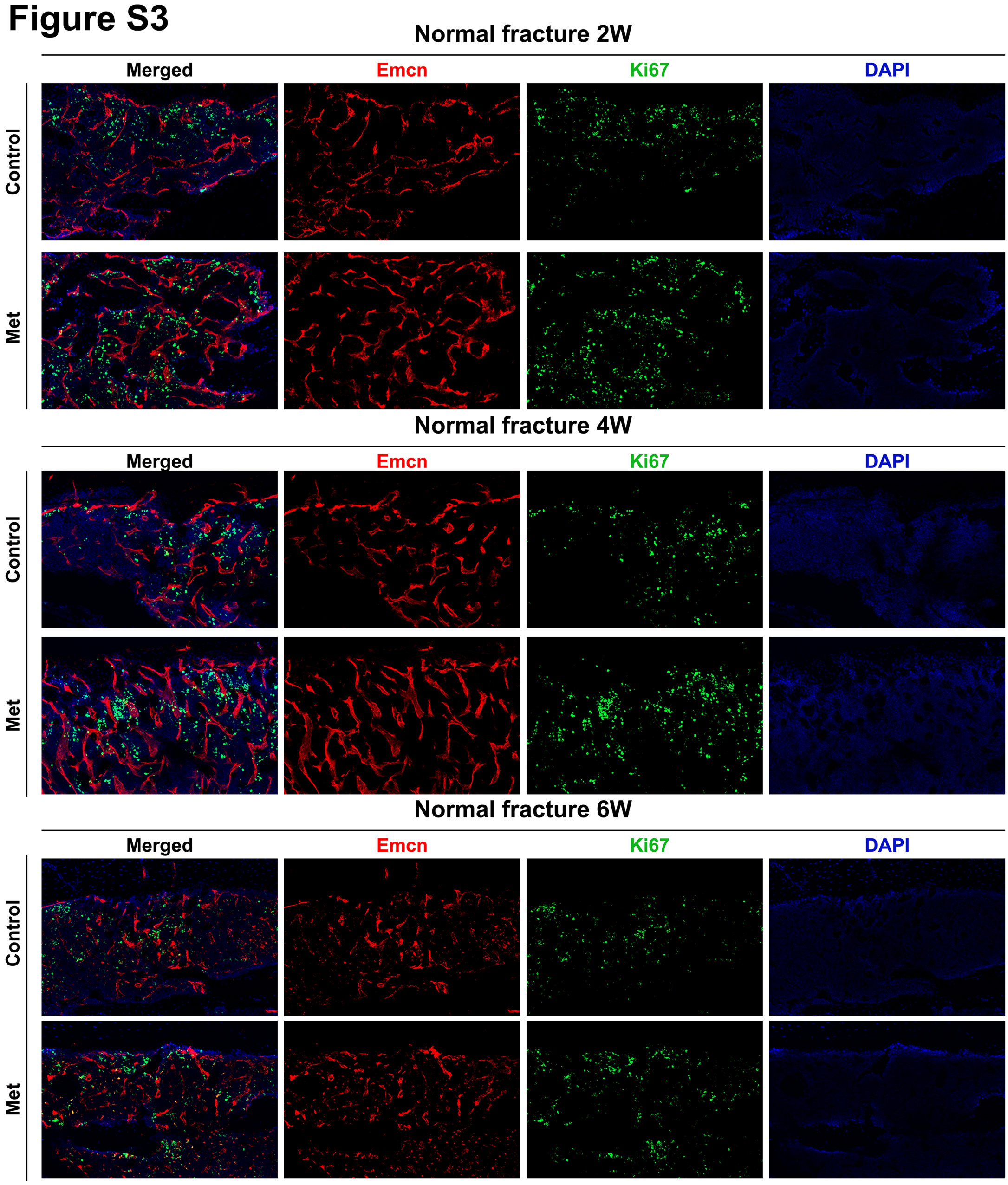
Normal fracture 2W
Merged
Emcn
Ki67
DAPI
Control
Met
Normal fracture 4W
Merged
Emcn
Ki67
DAPI
Control
Met
Normal fracture 6W
Merged
Emcn
Ki67
DAPI
Control
Met

Figure S4

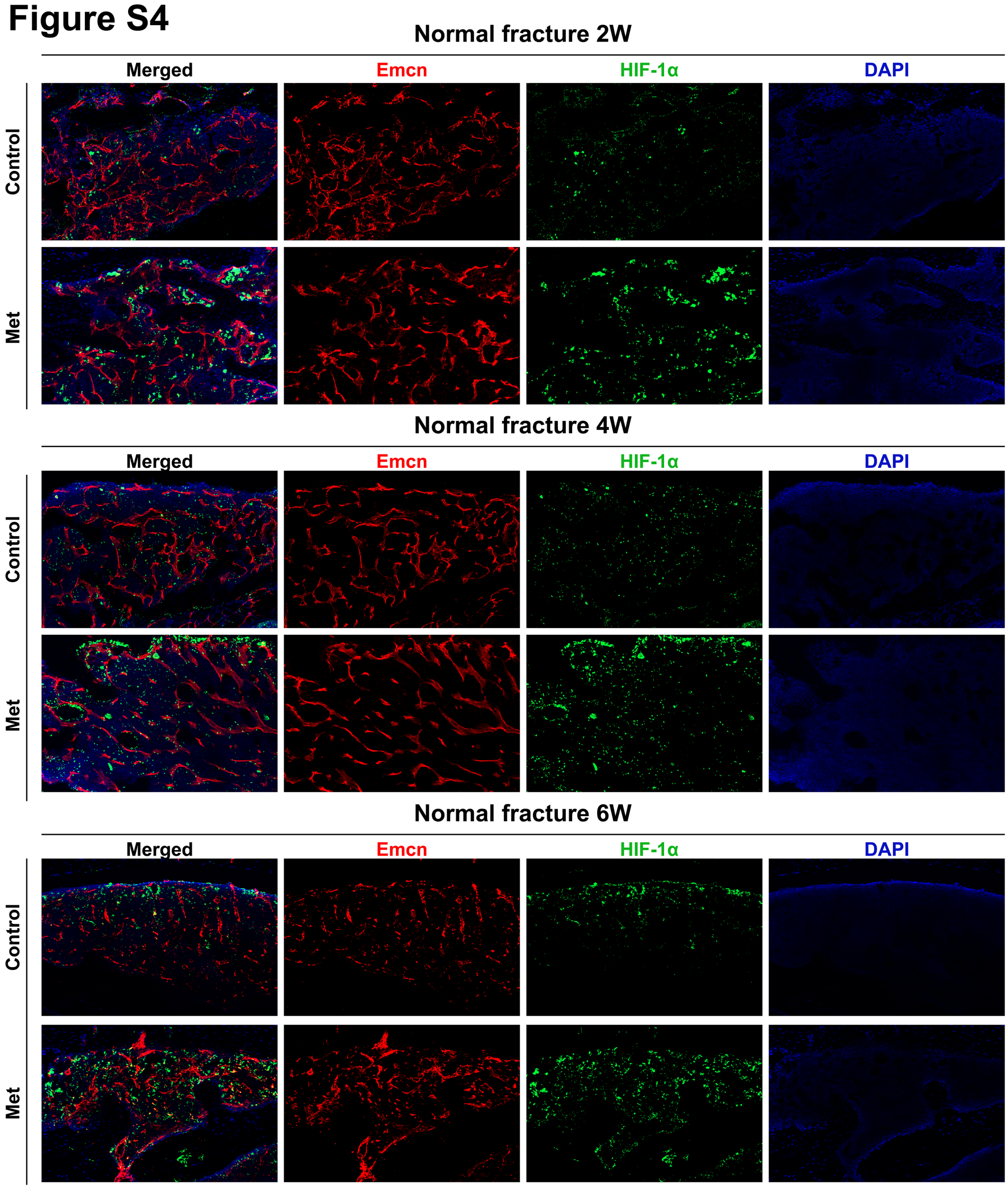

Normal fracture 2W
Merged
Emcn
HIF-1α
DAPI
Control
Met
Normal fracture 4W
Merged
Emcn
HIF-1α
DAPI
Control
Met
Normal fracture 6W
Merged
Emcn
HIF-1α
DAPI
Control
Met

Figure S4

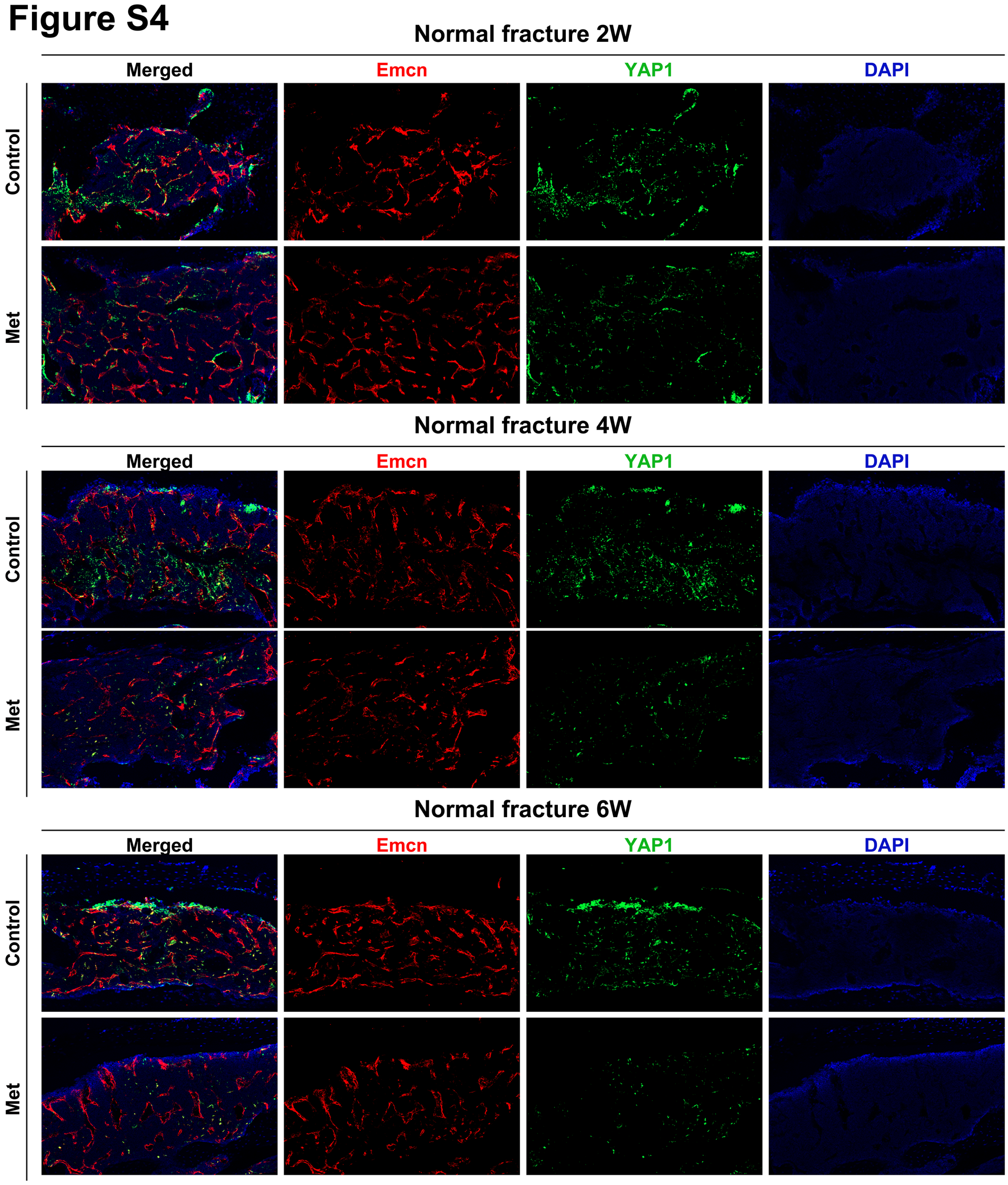
Normal fracture 2W
Merged
Emcn
YAP1
DAPI
Control
Met
Normal fracture 4W
Merged
Emcn
YAP1
DAPI
Control
Met
Normal fracture 6W
Merged
Emcn
YAP1
DAPI
Control
Met

Figure S4

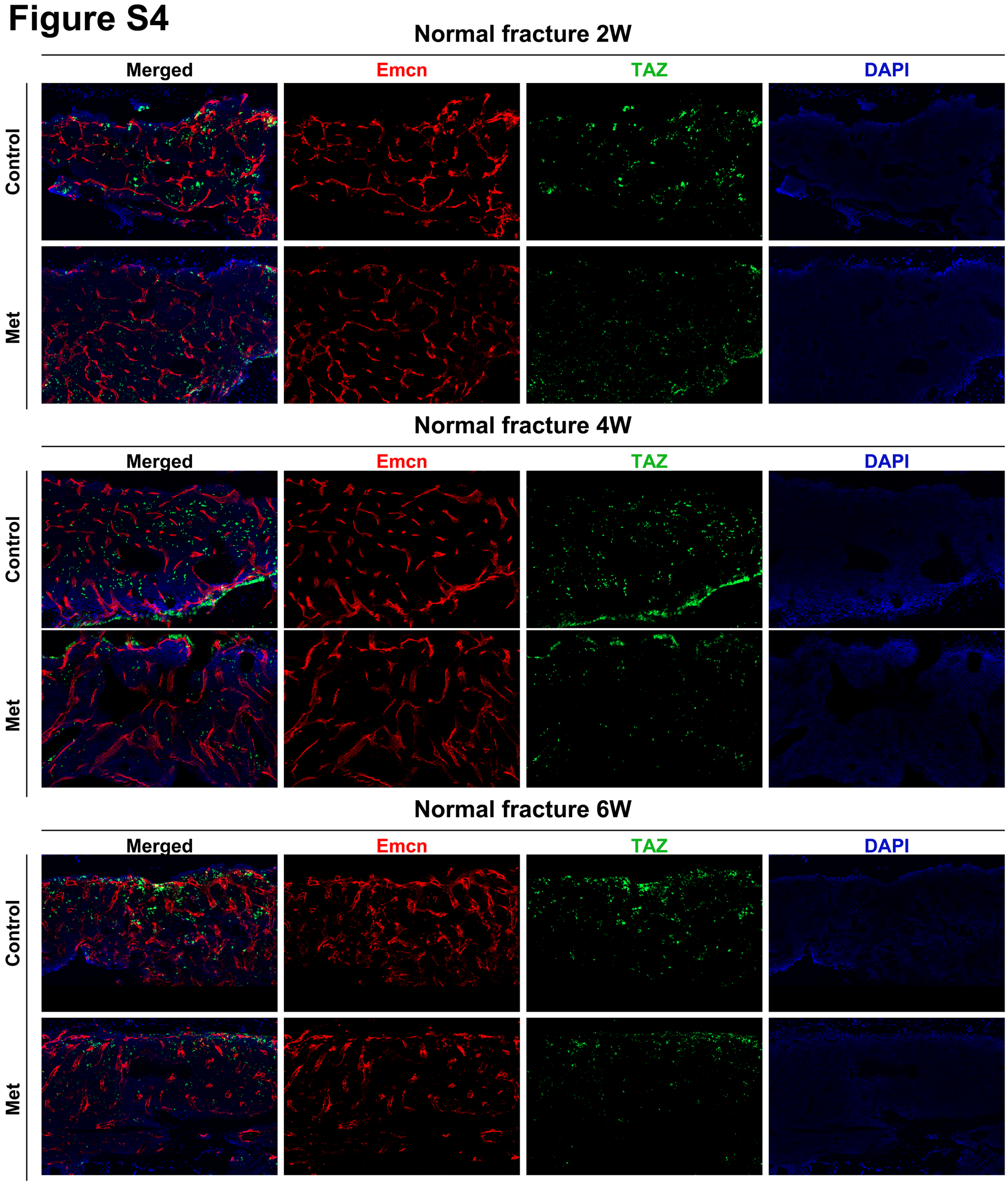
Normal fracture 2W
Merged
Emcn
TAZ
DAPI
Control
Met
Normal fracture 4W
Merged
Emcn
TAZ
DAPI
Control
Met
Normal fracture 6W
Merged
Emcn
TAZ
DAPI
Control
Met
